# Response of the wheat mycobiota to flooding revealed substantial shifts towards plant pathogens

**DOI:** 10.3389/fpls.2022.1028153

**Published:** 2022-11-28

**Authors:** Davide Francioli, Geeisy Cid, Mohammad-Reza Hajirezaei, Steffen Kolb

**Affiliations:** ^1^ Institute of Crop Science, Faculty of Agricultural Sciences, University of Hohenheim, Stuttgart, Germany; ^2^ Microbial Biogeochemistry, Research Area Landscape Functioning, Leibniz Center for Agricultural Landscape Research e.V. (ZALF), Müncheberg, Germany; ^3^ Department of Physiology and Cell Biology, Leibniz Institute of Plant Genetics and Crop Plant Research, Gatersleben, Germany; ^4^ Thaer Institute, Faculty of Life Sciences, Humboldt University of Berlin, Berlin, Germany

**Keywords:** wheat, flooding, mycobiota, metabarcoding analyses, fungal pathogens

## Abstract

Rainfall extremes are intensifying as a result of climate change, leading to increased flood risk. Flooding affects above- and belowground ecosystem processes, representing a substantial threat to crop productivity under climate change. Plant-associated fungi play important roles in plant performance, but their response to abnormal rain events is unresolved. Here, we established a glasshouse experiment to determine the effects of flooding stress on the spring wheat-mycobiota complex. Since plant phenology could be an important factor in the response to hydrological stress, flooding was induced only once and at different plant growth stages, such as tillering, booting and flowering. We assessed the wheat mycobiota response to flooding in three soil-plant compartments (phyllosphere, roots and rhizosphere) using metabarcoding. Key soil and plant traits were measured to correlate physiological plant and edaphic changes with shifts in mycobiota structure and functional guilds. Flooding reduced plant fitness, and caused dramatic shifts in mycobiota assembly across the entire plant. Notably, we observed a functional transition consisting of a decline in mutualist abundance and richness with a concomitant increase in plant pathogens. Indeed, fungal pathogens associated with important cereal diseases, such as *Gibberella intricans*, *Mycosphaerella graminicola*, *Typhula incarnata* and *Olpidium brassicae* significantly increased their abundance under flooding. Overall, our study demonstrate the detrimental effect of flooding on the wheat mycobiota complex, highlighting the urgent need to understand how climate change-associated abiotic stressors alter plant-microbe interactions in cereal crops.

## Introduction

Intense and long-lasting precipitations are expected to increase in frequency in Europe and other regions with ongoing global warming, which will in turn increase the frequency of flooding events (Tabari, 2020). Flooding causes a saturation of soil pore volume (i.e., waterlogging) and thus, gas transport including oxygen is substantially reduced. Cereal crop fitness and productivity are severely affected by water flooding since oxygen depletion occurs (Rhine et al., 2010; Morton et al., 2015; Ding et al., 2020), and even short-term flooding events (e.g., a few days) can significantly impact wheat growth (Malik et al., 2002). Wheat yield losses due to flooding and waterlogging range from 10% to over 50% (Jincai et al., 2001; Kaur et al., 2020), but they depend on stress duration, wheat genotype, growth stage, agricultural management, and soil characteristics (Kaur et al., 2020).

Flooding alters soil physiochemical properties, such as pH, redox potential, nutrient concentrations, and promotes oxygen depletion. Collectively, these changes in soil adversely affect the capability of a crop plant to survive (Visser et al., 2003; Dat et al., 2004; Niu et al., 2014). Equally important, flooding influences plant-microbe interactions, causing substantial compositional shifts of the plant microbiota with crucial consequences on its beneficial functionalities for the host plant (González Macé et al., 2016; Francioli et al., 2021a).

The biodiversity of soil-inhabiting fungi plays a key role in crop production and agricultural ecosystem functioning, especially in cereal cropping systems. Soil moisture is a key factor controlling fungal abundance and mycobiota structure. Most research on flooding and waterlogging of soil mycobiota has focused on wetlands, and thus, there is only a rather limited understanding of how the crop mycobiota is affected by waterlogging. Arbuscular mycorrhizal fungi (AMF) can support the growth and yield of crops by increasing mineral nutrient uptake, disease resistance and abiotic stress tolerance of crop plants, including cereals (Pellegrino et al., 2015). Plant benefits from mutualistic fungi may be negatively affected by soil waterlogging through the reduction of the initiation of mycorrhizal colonization of the host plants (Miller and Sharitz, 2000; Wolfe et al., 2006; Wang et al., 2016), which in turn reduces plant nutrition, in particular root phosphorous uptake (Deepika and Kothamasi, 2015). Flooding may also increase plant susceptibility to pathogens (Kirkpatrick et al., 2006), since infection by a number of pathogens is favored under anoxic conditions (Velásquez et al., 2018). On the other hand, flooding induces general defense pathways that may increase pathogen resistance and plant fitness (Adams et al., 2017).

Since the frequencies and intensities of extreme precipitation events are predicted to increase in the upcoming decades, it is crucial to understand how such environmental changes will affect the biodiversity and functions of fungal populations interacting with cereal crop hosts. Hence, we set up a pot experiment to explore the effects of flooding on wheat plant-mycobiota. In this experiment, flooding stress was induced only once, either at tillering, booting or flowering because plant phenology is an important driver in plant microbiota assembly (Donn et al., 2015; Francioli et al., 2018; Lewin et al., 2021) and abiotic stress may differentially affect the microbiota assembly dynamics depending on the specific plant growth stage (PGS) in which it occurs (Na et al., 2019; Breitkreuz et al., 2020). We used a metabarcoding approach to assess the response of the mycobiota associated with different plant compartments (phyllosphere, rhizosphere and root) to flooding stress. Several soil and plant parameters were measured to correlate physiological plant and edaphic changes with shifts in mycobiota structure. We expected that plant biomass would be negatively affected and that soil physico-chemical parameters and plant physiological state would change in response to flooding. Thus, we hypothesized that (i) the wheat mycobiota structure would be differentially affected depending on the timing of flooding events, with early mycobiota being more susceptible to community disruption. Furthermore, we hypothesized (ii) that these shifts in mycobiota assemblage between control and flooding treatment would be strongly correlated by alterations in the soil and plant traits induced by flooding stress. Lastly, we hypothesized (iii) substantial shifts in wheat mycobiota functional guilds as a response to flooding and waterlogging stress.

## Materials and methods

### Experimental setup

We investigated the response of the wheat mycobiota complex to flooding stress establishing a pot experiment that was conducted from September to December 2019 in a glasshouse at the Leibniz Institute of Plant Genetics and Crop Plant Research (IPK) in Gatersleben, Germany. Detailed description of the experimental setup is provided in Francioli et al. (2021a). In brief, seeds of spring wheat (*Triticum aestivum* L. Chinese Spring) were germinated in sieved soil (2 mm), which was obtained from the “Experimental Station Dedelow” (Prenzlau, Germany). The soil is classified as a sandy loam and its physico-chemical parameters are listed in **Table S1**. In the third week after sowing, seedlings were individually transferred to 10 L pots containing 5 kg of the soil used for germination (one seedling per pot). Wheat plants were grown under controlled conditions of day/night temperature, i.e., 18/16°C, air humidity 70%, light intensity 250-300 µE and photoperiod of 16 h light and 8 h darkness. A completely randomized design was used to place the pots on glasshouse tables. To monitor the developmental stage of the plants and the consequent flooding induction, we used the Zadoks scale (Zadoks et al., 1974). Flooding stress was induced only once and for a period of 12 days at tillering, booting, or flowering, and replicates were destructively sampled (**Figure S1**). Considering that the aim of the experiment was to investigate the response of the soil-wheat-mycobiota complex to severe water stress, flooding was induced for a period of 12 days to ensure sufficient oxygen depletion in the flooded treatments. Previous studies have shown that complete oxygen depletion in the top soil occurs within 2-8 days of flooding across a wide range of soils (Cannell et al., 1980; Meyer et al., 1985; Drew, 1992). Six replicates were established for each combination of plant growth stage and water treatment, for a total of 36 pots. Control plants were monitored at 50% WHC, which corresponded with the field capacity of the soil used in this study. Flooding treatment was established by keeping manually the water level at least 5 cm above the soil surface for 12 days.

On the twelfth day of exposition to flooding, and in the corresponding developmental stages, control and flooded plants were harvested, and tillers and spikes number recorded. Leaf material was collected only from fully expanded leaves, while rhizosphere soil was collected by manually uprooting wheat plants and shaking off the root-adhering soil into sterile zip bags. Afterward, roots were carefully washed with tap water to remove the remaining soil particles as much as possible. Soil, leaf and root samples were immediately frozen and stored at -80°C. Macro- (C, N, P, Mg, S, K and Ca) and micronutrient (Mn, Zn and Na) concentrations in the roots and leaves were measured using sector field high-resolution mass spectrometry (HR)-ICP–MS (Element 2, Thermo Fisher Scientific, Germany). Several edaphic parameters were also measured from the rhizosphere soil samples. Briefly, total soil organic carbon (TOC) and total nitrogen (TN) contents were determined in triplicate by dry combustion using a Vario EL III C/H/N analyzer (Elementar, Hanau, Germany). Plant available P (PDL) was extracted from fresh soil with double lactate extraction (1:50 w/v, pH 3.6, 1.5 h; Riehm (1943)). After filtration of the suspension (Whatman Schleicher and Schuell 595 1/5 Ø 270 mm), the extracted P was quantified colorimetrically using the molybdenum blue method (Murphy and Riley, 1962). Mn, Ca, Na, K, and Mg concentrations in soil were determined using inductively coupled plasma-optical emission spectrometry-ICP-OES (ICP-iCAP 6300 DUO, ThermoFisher Scientific, Germany). Although some soil and plant data have been published in a previous work (Francioli et al., 2021a), here we present the full dataset of soil and plant properties measured in the study.

### DNA extraction, amplicon library preparation and sequencing

DNA was extracted from the collected material using respectively 0.35g of soil, leaf and root, with the DNeasy PowerLyzer PowerSoil Kit (Qiagen). We employed the same DNA extraction kit for the isolation of the genomic material from all the collected samples to allow the comparison of fungal communities across compartments, as suggested in Francioli et al. (2021b). Fungal DNA amplification was performed using the primers ITS1F/ITS2R (White et al., 1990) using the following PCR protocol: PCR was carried out in a 50 μl reaction volume with 1 μl of DNA template (~ 5ng), 0.2 mM dNTPs and 0.4 μM of each primer (PCR conditions: 95°C for 5 min; 35 cycles at 95°C for 1 min, 56°C for 1 min and 72°C for 1 min; and 72°C for 5 min). The amplicons were sent to LGC Genomics GmbH (Berlin, Germany) for barcoding and paired-end sequencing on Illumina MiSeq v3 platform. Demultiplexing was performed using Illumina bcl2fastq 2.17.1.14 software following clipping of barcode and sequencing adapters. Primers were removed using Cutadapt v3.4 (Martin, 2011) following sequence processing using QIIME 2 v2022.2 (Bolyen et al., 2019). Denoising was performed by using the build-in method for DADA2 (Callahan et al., 2016) with forward and reversed reads truncated at 250 bp and 220 bp, respectively. The DADA2 pipeline started from 13,427,188 reads and yielded 9,005,083 non chimeric sequences. Amplicon sequencing variants (ASV) produced by DADA2 were assigned to taxonomy using the naïve bayesian classifier (Wang et al., 2007) against the Unite 8.3 reference database (Nilsson et al., 2018), and non-fungal ASVs were discarded. Only ASVs that were detected in more than two samples were included in the data analyses. Alpha diversity metrics were calculated from the normalized sequence library, which was rarefied to 20,000 reads per sample.

### Functional characterization of the fungal ASVs

We characterized the ASV data into three functional guilds, pathogens, saprotrophs, and mutualists, based on functional guilds associated with a given taxonomic level reported in the databases FUNGuild (Nguyen et al., 2016) and FungalTraits (Põlme et al., 2020) according to the authors’ instructions. To create the subset of pathogenic ASVs, we followed the procedure described in Francioli et al. (2020). Briefly, we kept only the identified pathogen ASVs that were taxonomically characterized at the species level, and then their plant pathogenicity was cross-checked using the literature references (Agrios, 2004; Domsch et al., 2007; Arnolds and van den Berg, 2013; Farr and Rossman, 2014; Dighton, 2016; Dighton and White, 2017) to include pathogens that are associated with well-established plant diseases. We acknowledge that the *modus operandi* used to attribute the “pathogen” classification to the fungal species identified may have introduced some biases, since the effective pathogenicity of a particular fungal taxon also depends on the realized host-fungus interactions and the environmental context (van Ruijven et al., 2020; Ampt et al., 2022). By comparison, the classification of the identified ASVs in saprophytic and mutualistic fungi was less complicated. Fungal saprobes are merely those that have only been reported as free-living or in combination with an endophytic guild, whereas mutualistic fungi were those taxa reported as arbuscular mycorrhizal (obligate symbionts) or exclusively endophytic fungi (i.e., fungal endophytes that have not been reported as pathogenic or saprotrophic) (Lozano et al., 2021). In total, 502 fungal ASVs were assigned to a functional guild, representing 42.7% of the total fungal sequences.

### Statistical analyses

Differences in soil and plant properties were tested among the treatments and plant growth stage (PGS) by univariate analysis of variance (ANOVA) followed by Tukey’s honestly significant difference (HSD) *post hoc* test. All variables included in the analysis were tested for normality using Shapiro-Wilk and Jarque-Bera tests, and the homogeneity of variance was examined using Levene’s test. A log10 transformation was applied to all variables that did not meet the parametric assumptions. Univariate PERMANOVA models were used to test the effects of soil-plant compartment, PGS and watering treatment on fungal richness (Anderson, 2017). Pairwise differences in fungal richness between watering treatments at the same PGS and compartment were estimated using ANOVA followed by Tukey’s HSD *post hoc* test. Differences in the fungal community structure were determined across plant-soil compartments, PGSs and flooding treatments. We first calculated Bray-Curtis dissimilarities using Hellinger transformation (square root transformation of relative abundances; Legendre and Gallagher (2001)). Permutational multivariate analysis of variances (PERMANOVA) based on Bray-Curtis dissimilarity was performed to analyze the effect of the abovementioned experimental factors on the mycobiota structure using 999 permutations for each test. Structural dissimilarities of the mycobiota between flooding and control treatments at each PGS were compared to resolve at which PGS the application of flooding had the largest effect. Variance partitioning based on redundancy analysis (RDA) was performed to quantify the contribution of soil properties, plant attributes, PGSs and watering treatments to the structure of fungal communities in each compartment. Following Blanchet et al. (2008), the significance of the global model using all predictors was tested first. Variable selection was performed using forward selection implemented with the *forward.sel* function in the R package “packfor” (Dray et al., 2011). Variance partitioning was conducted using the *varpart* function in the “vegan” R package (Oksanen et al., 2018). We then constructed a model of multivariate analysis of variance using distance-based redundancy analysis (db-RDA) based on the Bray-Curtis distance to determine the environmental variables that were most influential on the mycobiota structure of each plant compartment. Fungal biomarker taxa were identified by explaining differences between the mycobiota compartments and between flooding and control treatments at each PGS in all three plant-soil compartments, employing a linear discriminant analysis effect size (LEfSe) (Segata et al., 2011). To test whether the relative abundance of a specific fungal taxa or the cumulative abundances of the three fungal guilds (pathogens, saprobes and mutualists) were affected by watering treatment we built factorial GLMs with negative binomial errors, building a separate model for each test using the *glm.nb* function in the “MASS” R package (Venables and Ripley, 2002). All data were analyzed with R version 4.0 (R Core Team, 2020).

## Results

### Effect of flooding on plant performance, plant traits and on soil properties

Flooding had a detrimental effect on wheat fitness. We found a reduced root biomass (55%) at tillering (*P<* 0.05), while at booting, shoot and root dry biomass decreased by 25% and 70%, respectively (**Table S2**). Furthermore, significant (*P<* 0.05) decreases in the number of tillers (29%) and spikes (13%) were associated with flooded wheat plants (**Table S2**). Significant effects of flooding on the measured soil-plant traits were widely observed across all plant compartments. Overall, significant increases (*P<* 0.05) in soil moisture, pH, Zn, and available P was observed in all flooded soil samples (**Table S3**). Flooding had also a severe effect on root and leaf properties, as the concentration of all the plant nutrients measured were strongly affected by this stressor, especially at early stage of plant growth. For example, the root and leaf N, S, P, Mg and K concentrations were significantly (*P<* 0.05) lower in the flooded wheat plants at tillering and booting stage (**Table S3**). On the contrary, total soil C and S, root S and Na, and leaf C showed a significant (*P<* 0.05) different trend, being higher (*P<* 0.05) in the control than in the flooded samples.

### Effect of plant compartment, PGS and flooding on the wheat mycobiota

**Figure 1:**
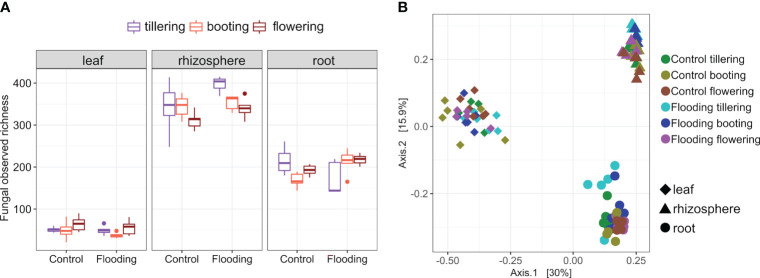
Characteristics of the wheat mycobiota. **(A)** Observed richness and **(B)** principal coordinate analysis of the wheat mycobiota across plant compartment, plant growth stage and watering treatment.

**Figure 2:**
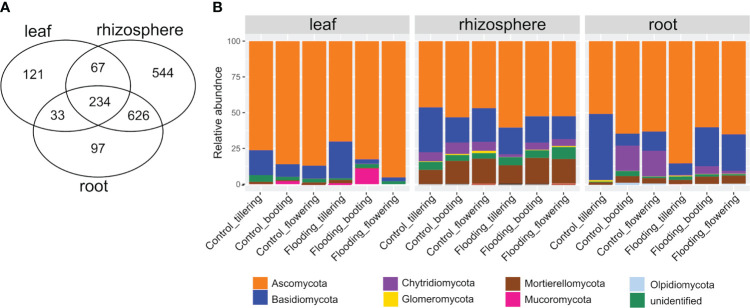
Composition of the wheat mycobiota. **(A)** Venn diagram showing the shared and unique fungal ASVs between soil-plant compartments. **(B)** Relative abundance of the main fungal phyla composing the wheat mycobiota across plant compartment, plant growth stage and watering treatment.

The ITS rRNA gene high-quality reads recovered from all samples clustered in 1772 fungal ASVs. Fungal richness ranged from 21 to 415 ASVs and differed significantly among plant compartments (P< 0.001, **Table S4**), with the rhizosphere having the highest and the phyllosphere the lowest number (**Figure 1A**). Compartmentalization explained most variation in total fungal richness, whereas plant growth stage (PGS) and watering treatment (WT) had a marginal effect (**Table S4**). Plant compartment was also the main factor for variation in mycobiota structure (44% of variance; **Table 1**), and principal coordinate analysis (PCoA) clearly reflected this finding (**Figure 1B**). Looking belowground, the root-associated fungal (RAF) ASVs were mainly a subset of the rhizosphere community (**Figure 2A**). However, large differences in the abundances of the dominant fungal taxa were observed between these two belowground compartments. For instance, the rhizosphere mycobiota was characterized by a significantly higher proportion (*P<* 0.05) of the phyla Mortierellomycota, Chytridiomycota, and Zoopagomycota and of the class Tremellomycetes (Basidiomycota) compared with the other compartments (**Figure S2**). On the other hand, wheat roots were enriched (*P<* 0.05) in fungal taxa affiliated with the Basidiomycota classes Agaricomycetes and Cystobasidiomycetes. Leaf mycobiota was primarily composed by taxa within the Ascomycota classes Dothideomycetes and Sordariomycetes (**Figure S2**). Notably, taxa associated with the phyla Olpidiomycota and Mucoromycota were solely identified in root and leaf samples, respectively (**Figure 2B**).

**Table 1:** The effect of the soil-plant compartment, plant growth stage (PGS) and watering treatment (WT) on the wheat mycobiota structure.

Parameter	df	Pseudo-F	R^2^	P‐value
Compartment	2	52.276	0.447	0.001
PGS	2	5.120	0.044	0.001
WT	1	3.048	0.013	0.008
Compartment * PGS	4	3.559	0.061	0.001
Compartment * WT	2	2.671	0.023	0.003
PGS * WT	2	2.277	0.019	0.015
Compartment * PG * WT	4	1.960	0.034	0.004

**Figure 3:**
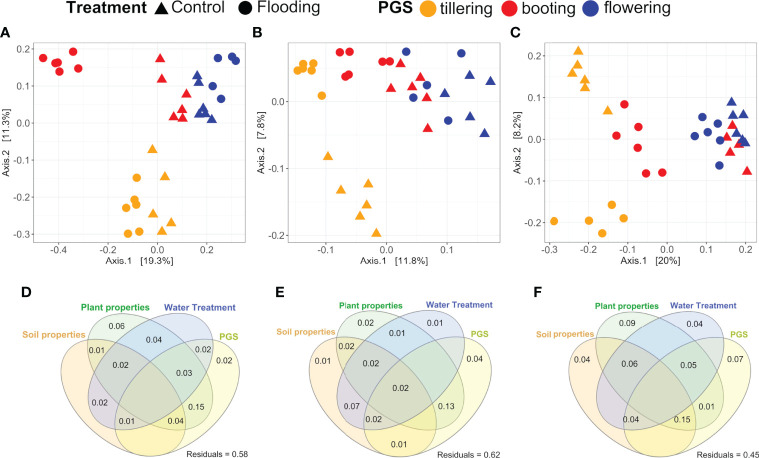
Effect of the experimental variables on the spring wheat-mycobiota complex. Principal coordinates analysis (PCoA) of the mycobiota detected in the **(A)** leaf, **(B)** rhizosphere and **(C)** root compartments. Variance partitioning analysis illustrating the effects of soil parameters, plant traits, watering treatment and plant growth stage (PGS) on the mycobiota associated with the **(D)** leaf, **(E)** rhizosphere and **(F)** root compartments.

Within each compartment, substantial and significant effects of flooding and plant phenology on the mycobiota structure were found. In general, flooding explained approximately 7% of the variance across all plant compartments, while PGS accounted for 20% of the variation in roots and leaves and 14% in the rhizosphere (**Table 2**). We detected a significant interaction between PGS and WT, which explained an additional 7.2%, 10.8% and 9.8% of variation in the rhizosphere, root and leaf, respectively. This interaction suggests a differential response of the wheat mycobiota to flooding, which is, however, dependent on the PGS at which flooding stress was induced. Principal coordinates analysis of each investigated compartment confirmed the PERMANOVA results, distinguishing the samples associated with a particular PGS along the first axis, while the second coordinate clearly separated flooded samples from the corresponding controls (**Figures 3A–C**).

**Table 2:** The effect of plant growth stage (PGS) and watering treatment (WT) on the fungal community structure associated with the rhizosphere, root and leaf compartments.

Parameter	df	Pseudo-F	R^2^	P‐value
*Rhizosphere*				
PGS	1	2.712	0.14	0.001
WT	2	2.534	0.065	0.001
PGS * WT	2	1.4	0.072	0.007
*Root*				
PGS	1	4.375	0.197	0.001
WT	2	2.966	0.067	0.001
PGS * WT	2	2.399	0.108	0.001
*Leaf*				
PGS	1	4.801	0.213	0.001
WT	2	2.986	0.066	0.001
PGS * WT	2	2.206	0.098	0.001

**Figure 4:**
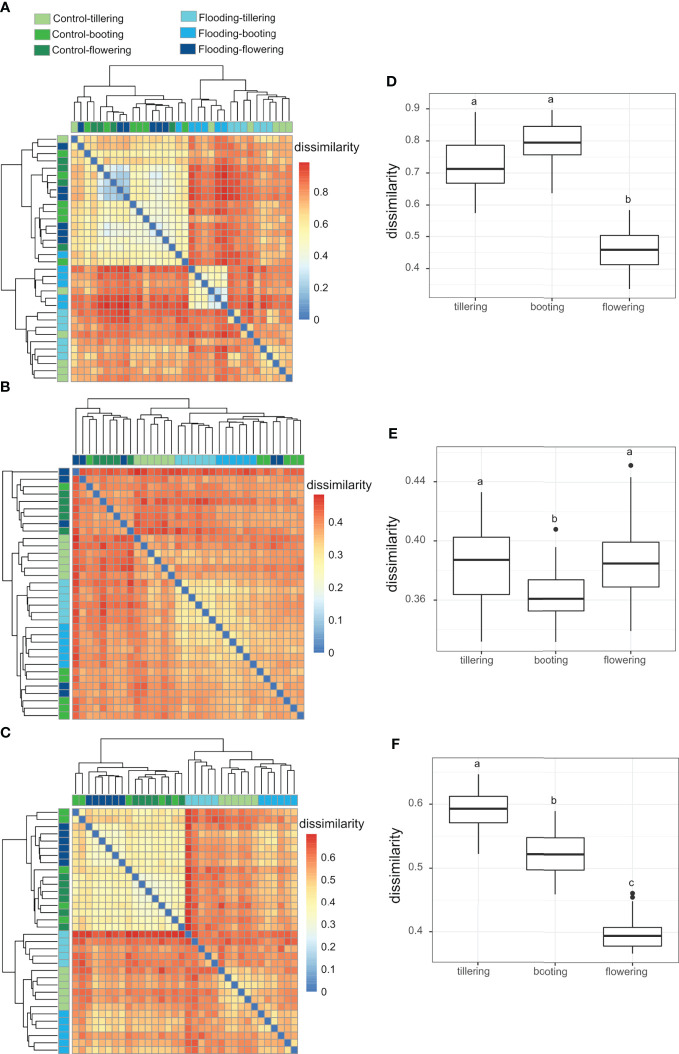
Heatmap representing the Bray-Curtis dissimilarity of mycobiota structure between watering treatment and plant growth stage (PGS) in the **(A)** leaf, **(B)** rhizosphere and **(C)** root compartments. Box plots of the Bray-Curtis dissimilarities between flooding and control samples at each PGS in the **(D)** leaf, **(E)** rhizosphere **(F)** and root compartments. The different letters indicate significant differences among PGS (Tukey’s HSD test P< 0.05).

To understand at which PGS the application of flooding had the largest effect on the mycobiota assemblage dynamics, we compared structural dissimilarities of the fungal communities between flooding and control treatments at each PGS. In the rhizosphere, the largest impact of flooding stress on the mycobiota community assemblage was observed in the earliest (tillering) and latest stages (flowering) of plant growth (**Figure 4**). In the roots and leaves, the lowest impact of flooding on fungal community structure was observed at flowering, while the highest impact was observed at tillering and booting. LEfSe analysis confirmed that flooding stress caused a larger disruption to early (tillering) compared with late (booting and flowering) wheat mycobiota in all compartments, identifying at tillering always twice as many fungal biomarker taxa as in the other PGSs (**Figures S3-5**). Furthermore, mycobiota dissimilarities between flooding and control treatments were always lower in the soil than in the leaves and roots (**Figure 4**), suggesting that the effects of flooding on fungal community assembly were more pronounced on plant-associated fungi.

Finally, variance partitioning was performed to quantify the contribution of soil and plant properties and their interactions with watering treatment and PGS on the structure of the wheat mycobiota. These four experimental factors captured a large proportion of the variance, accounting for 55%, 42% and 38% in the root, leaf and rhizosphere, respectively (**Figures 3D–F**). Within the rhizosphere and leaf compartments, the pure effect of these variables on the wheat mycobiota structure was marginal, since most variance explained by them was shared. In contrast, the root mycobiota was considerably influenced by the pure effect of plant traits (9% of variance), PGS (7% of variance) and WT (4% of variance) and by the interactions of these factors and soil parameters (>20% of variance). These findings suggested an important interactive effect of PGS and WT on plant and soil properties, which in turn significantly affected mycobiota assembly. db-RDA further revealed that root C and Mn together with leaf N, Mg and Mn content were significant factors (*P<* 0.05) affecting RAF assembly (**Table 3**). The fungal community inhabiting the rhizosphere was mainly influenced by soil parameters such as pH, soil K and PDL (*P<* 0.05), together with root N and Na concentration. On the contrary, the leaf mycobiota was significantly (*P<* 0.05) affected primarily by leaf traits as Na, Mg, Mn and K concentration, and by root K and root Mn (**Table 3**).

**Table 3:** Relationships between the predictor soil and plant properties and the mycobiota in the leaf, rhizosphere and root compartments.

	Leaf		Rhizosphere		Root	
	F	P	F	P	F	P
Soil pH	ns	ns	**2.642**	**0.001**	ns	ns
PDL	ns	ns	**1.525**	**0.013**	ns	ns
Soil S	ns	ns	**1.336**	**0.049**	ns	ns
Root C	ns	ns	**1.364**	**0.045**	**1.429**	**0.05**
Root N	ns	ns	**1.420**	**0.018**	ns	ns
Root Na	ns	ns	**1.341**	**0.041**	ns	ns
Root K	**1.647**	**0.002**	ns	ns	ns	ns
Root Mn	**1.613**	**0.004**	ns	ns	**2.471**	**0.001**
Leaf N	ns	ns	ns	ns	**2.027**	**0.002**
Leaf Na	**5.501**	**0.001**	ns	ns	ns	ns
Leaf Mg	**4.821**	**0.001**	**3.550**	**0.001**	**7.330**	**0.001**
Leaf Mn	**2.566**	**0.001**	ns	ns	ns	ns
Leaf K	**1.588**	**0.005**	ns	ns	**1.760**	**0.005**

The results show marginal tests using the db-RDA model. Significant P values less than 0.05 are indicated in bold. ns, not significant.

### Pathogens

**Figure 5:**
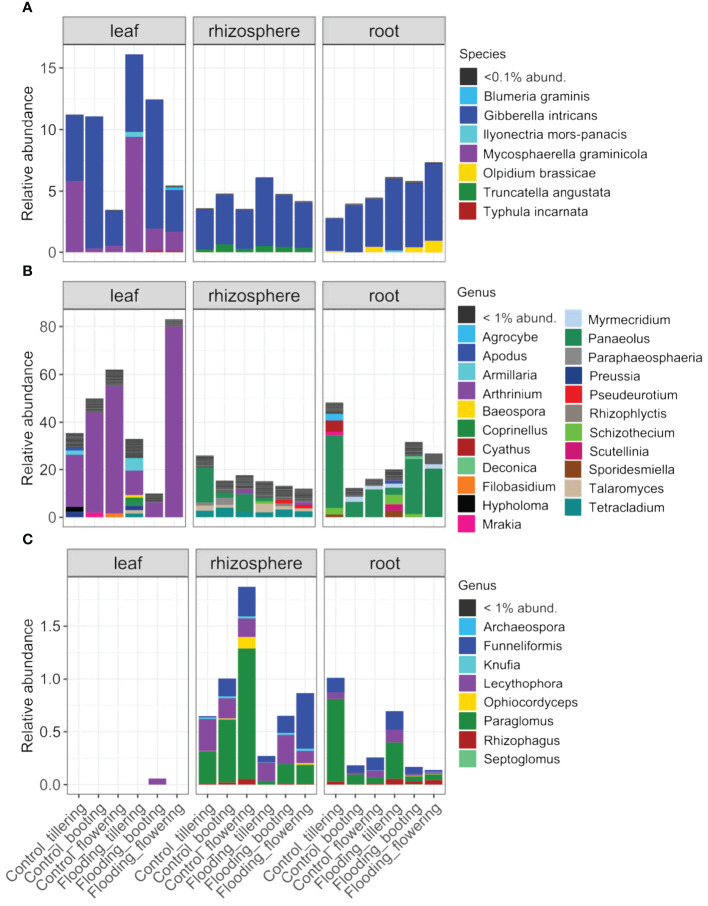
Relative abundance of the **(A)** pathogenic, **(B)** saprotrophic and **(C)** mutualistic taxa detected in the different compartments, plant growth stages and watering treatments.

We identified 28 ASVs from the 1772 fungal ASVs that are known as plant pathogenic fungal taxa (**Table S5**). They accounted for 6.5% of the total fungal reads and were affiliated with 16 fungal species, mainly of the genera *Gibberella*, *Olpidium*, *Mycosphaerella*, *Ilyoectria* and *Typhula*. Belowground compartments were characterized by distinct pathogenic taxa and by a significant (*P<* 0.05) higher number of pathogens, while PSG and WT had a marginal or no effect on pathogen richness (**Table S6**; **Figure S6**). Flooding notably influenced plant fungal pathogenic community assembly (**Figure S7**; **Table S7**), since it significantly (*P<* 0.05) increased their abundance (from 5.4% to 7.6% of total fungal reads in the control and flooding samples, respectively), and particularly at the early stage of plant growth (**Figure 5A**). *Gibberella intricans* (teleomorph of *Fusarium equiseti*), the causal agent of head blight and crown rot in cereals, was (i) the most representative pathogen identified (accounting for 5.1% of total fungal sequences), (ii) detected in all samples, and (iii) significantly (*P<* 0.05) more abundant in flooding (5.8% of total reads) than in the control treatments (4.4%). Most other representative fungal pathogens were associated with a specific compartment. This was the case for *Mycosphaerella graminicola*, the causal agent of wheat leaf blotch, which was exclusively detected in wheat leaves and showed a significant increase (*P<* 0.05) in all flooded plants compared with the control plants (**Figure 5A**). Similarly, *Typhula incarnata*, a fungal species responsible for Typhula blight in wheat (Lawton and Burpee, 1990), was uniquely found in the flooded rhizospheric samples. *Olpidium brassicae*, a soil-borne root-infecting pathogen (Hartwright et al., 2010) and a vector of plant viruses (Campbell, 1996) that has been previously reported in wheat roots (Esmaeili Taheri et al., 2015), was solely detected in root samples. *O. brassicae* was also found significantly more abundant (*P*< 0.05) in flooded roots and was mainly associated with late PGSs, such as booting and flowering (**Figure 5A**).

### Saprotrophs

Saprotrophs were represented by 394 ASVs and accounted for a large proportion of the mycobiota, ranging from 25% in the belowground compartments to more than 50% in the phyllosphere (**Figure 5B**). The saprophytic communities differed significantly in structure, composition and richness between compartments and responded significantly to WT and PGS (**Figures S8-9**; **Table S8-S9**). *Panaleous fimicola*, a ubiquitous soil saprotroph belonging to Agaricomycetes, was the most abundant saprobe found belowground (accounting for 7.4% of total fungal sequences), but it was not detected in the phyllosphere. *P. fimicola* was significantly influenced by WT, as it was almost depleted in rhizosphere samples under flooding (from 8.1% to 0.8% of total fungal reads in the control and flooding samples, respectively) (**Figure 5B**). The rhizosphere mycobiota was characterized by a proportion of saprotrophic taxa affiliated with the cosmopolitan Ascomycota genera *Tetracladium* and *Talaromyces*, which were scarcely detected in the other compartments. In the roots, we found coprophilous taxa associated with the Sordariomycetes genera *Myrmecridium* and *Schizothecium*. Their abundance shifted across PGS, regardless of WT. The saprophytic community of the phyllosphere was structured by completely different fungal taxa, with large compositional shifts across PGS and WT (**Figure 5B**). *Arthrinium malaysianum* and *Preussia pilosella* were the most representative species, and they were not detected belowground. Furthermore, leaf-inhabiting endophytes of Basidiomycetous yeasts *Filobasidium*, which are endophytes and occur in the phyllospheres and grains of several wheat varieties (Nicolaisen et al., 2014; Sapkota et al., 2017), were uniquely found in leaves, and they were not affected by WT.

### Mutualists

Mutualists represented a low proportion of the total mycobiota, accounting for approximately 1% of the total sequences and 80 ASVs. Of these ASVs, 53 were identified as AMF species (phylum Glomeromycota, 0.56% of total fungal reads), and *Archaeospora trappei*, *Funnelifromis caledonium*, *Parglomus laccatum* and *Rhizophagus rregolaries* were the most abundant AMF identified. The remaining 17 ASVs corresponded to root endophytic fungi affiliated with the Ascomycota genera *Knufia* and *Lecythophora*. Mutualist richness depended on the plant compartment, since none were identified in the phyllosphere. Interestingly, mutualist richness and community structure were strongly affected by flooding (**Table S10-S11**, **Figure S10**), as their richness and abundance decreased dramatically in flooded soils and roots (**Figure 5C**, **Figure S11**). The deleterious effect of flooding on the mutualist taxa was more evident in the rhizosphere than in the roots (**Table S11**; **Figure 5C**). PGS had also a significant effect (*P<*0.05) on richness and structure of mutualistic fungi, which was more pronounced in the rhizosphere, as the abundance of these beneficial taxa consistently increased with plant maturity.

### Changes of soil and plant properties over PGS and WT significantly influenced fungal functional guilds

Variance partitioning and db-RDA model analysis revealed a strong contribution of soil and plant properties and their interactions with watering treatment and PGS on the structure of the characterized fungal functional guilds (**Figure S12**; **Table S12**). In general, the fungal guilds associated with roots and leaves were more affected by the experimental variables than those in the rhizosphere (**Figure S12**). For instance, variance partitioning captured more than 35% of the variation within the root and leaf pathogen communities, revealing that root pathogens were significantly (*P<* 0.05) correlated with soil pH, S and K, while phyllospheric pathogens were correlated with root C, root S, leaf Na and Mn contents (**Table S12**). As expected, the saprotrophic communities were significantly associated with specific properties characterizing the compartment in which they were detected. Indeed, the rhizosphere saprotrophs were significantly (*P<* 0.05) affected by soil pH and root C, Mn and Mg. Root saprobes were influenced by root C, P and Mg concentration, while leaf S, Na and C contents affected the phyllosphere saprophytic community (**Table S13**). Mutualistic taxa were solely identified belowground. The root mutualists correlated with root K and Na concentrations. Interestingly, the rhizospheric mutualistic community was associated with shifts in soil pH, PDL and root S content (**Table S14**).

## Discussion

Our study demonstrated clear detrimental effects of flooding on the spring wheat mycobiota complex. In addition to the negative impact of flooding on wheat fitness, dramatic compositional shifts in fungal communities were observed in the flooded samples across the three soil-plant compartments investigated. To a large extent, these differences were explained by the pure and interactive effects of flooding and plant growth stage (PGS) on plant and soil properties. Our work also highlighted that flooding can significantly restructure the wheat mycobiota, in particular altering the composition and abundance of agriculturally relevant taxa. Under flooding, the relative abundance of pathogenic fungi increased compared with the control plants, regardless of PGS and compartment. Conversely, mutualistic taxa, especially AMF, significantly decreased their richness and presence in all flooded samples. These findings support the idea that flooding represents a substantial threat to crop and cereal productivity under climate change. Therefore, it is imperative to unravel factors influencing the soil-plant-mycobiota complex and its functionalities in response to climate change-associated extreme weather events (Rahmstorf and Coumou, 2011; De Vries and Shade, 2013).

### Flooding reshaped the wheat mycobiota

Our study provided an in-depth characterization of the effects of flooding stress on the spring wheat mycobiota. The mycobiota was greatly affected by flooding, as distinct fungal taxa characterized the flooding and control samples across all three soil-plant compartments, rhizosphere soil, roots and leaves. In general, flooding stress caused large shifts in the mycobiota structure at each PGS it was induced. However, it had the greatest impact on the mycobiota assembly at tillering, especially in the root compartment. These findings acknowledged our first hypothesis, as they demonstrated that flooding caused a greater disruption to early compared with late PGS mycobiota. Recent studies have reported similar results, with the juvenile plant-associated microbiota more affected by water stress compared with the microbiota associated with late stages of plant development (Xu et al., 2018; Francioli et al., 2021a; Francioli et al., 2022). Taken together, these observations may imply that the mycobiota of young plants is still in a dynamic process of establishment, in which community assembly is less resilient to abiotic and biotic stresses. Hence, a more stable mycobiota can be expected to be associated with the plant during late growth stages by prior establishment of a more stable community, i.e., likely with a higher and tighter degree of interactions (Angel et al., 2016; Edwards et al., 2018; Lewin et al., 2021). Interestingly, we observed a more severe impact of flooding on the mycobiota associated with plant compartments (roots and leaves) compared with the rhizosphere compartment. This highlights the selective pressure exerted by the plant host, corroborating that community assembly dynamics of plant-associated fungi are to a large extent under host control (Sapkota et al., 2015; Agler et al., 2016). Moreover, our analysis showed that the spring wheat mycobiota, especially the root-associated mycobiota, was significantly correlated with plant traits and by their interactive effect with edaphic properties, PGS and WT. Indeed, flooding dramatically affected plant and soil properties, such as soil pH and many root and leaf attributes, which in turn were significantly associated with shifts in the mycobiota structure across the wheat plants (**Table 3**). Changes in soil pH are a commonly reported consequence of waterlogging (Sun et al., 2007; Hemati Matin and Jalali, 2017), and it has been recognized as a key driver in structuring the mycobiota across a wide range of soils and ecosystems (Fierer and Jackson, 2006; Lauber et al., 2009; Bardelli et al., 2017; Guo et al., 2020). Plant traits, such as root and leaf nutrient concentrations, have been widely described as important factors in shaping the plant mycobiota (Kembel and Mueller, 2014; Fitzpatrick et al., 2018; Freschet et al., 2021), and their variations may significantly impact community assembly (Leff et al., 2018; Ulbrich et al., 2021; Maywald et al., 2022). Collectively, these findings validated our second hypothesis, highlighting the detrimental influence of flooding on plant and soil properties, which in turn are strongly associated with mycobiota structure and assembly dynamics.

### Detrimental effects of flooding on agriculturally relevant fungal clades

Flooding resulted in a substantial restructuring of the plant-associated mycobiota along with a dramatic taxonomic and functional guild change, validating our third initial hypothesis. We observed a general and considerable decline in the relative abundance and richness of mutualists and a concomitant increase in the relative abundance and richness of pathogens and saprotrophs. Belowground, arbuscular mycorrhizal fungi (AMF) relative abundance declined in the flooded samples by nearly two-thirds across all plant stages, driven primarily by *Paraglomus* and *Funnelifromis* genera. These findings are in accordance with greenhouse (Miller and Sharitz, 2000; Deepika and Kothamasi, 2015) and field studies (Barnes et al., 2018) that observed reduced mycorrhizal hyphal growth and root colonization under high soil moisture content. The significant increase in plant available P measured in the flooded soils might also be linked to the reduction in AMF richness and abundance, since soil available P governs the level of root colonization by arbuscular mycorrhizal fungi in agro- and natural ecosystems (Verbruggen et al., 2013; Camenzind et al., 2014; Liu et al., 2016; Wang et al., 2017). Furthermore, mycorrhizal fungi may play a vital role in improving plant resistance and tolerance to biotic stressors such as pathogens (Pozo et al., 2009). Thus, the increased detection of pathogens under flooding might be a consequence of reductions in the diversity and composition of AMF and in their inability to colonize roots under flooding conditions (Azcón-Aguilar et al., 2002; Singh and Giri, 2017). In our study, fungal pathogens associated with important cereal diseases, such as *Gibberella intricans*, *Mycosphaerella graminicola*, *Typhula incarnata*, significantly increased their abundance under flooding, which further supports the detrimental effect of high soil moisture levels on the wheat mycobiota complex. Predictably, flooding also favored the enrichment of the aquatic fungus and root-infecting obligate plant parasite *Olpidium brassicae*, which was mainly observed in flooded roots at late PGSs (booting and flowering). These findings are in line with recent studies that have shown significant increases in the abundance and richness of fungal pathogens under flooding (Kirkpatrick et al., 2006; Barnes et al., 2018) and drought (Choudhary et al., 2016; Francioli et al., 2020; Lozano et al., 2021) across different ecosystems, indicating a strong linkage between pathogen abundance and reduced plant performance under these abiotic stresses (Chakraborty et al., 2000; Garrett et al., 2006). Interestingly, across all soil-plant compartments, the abundance of the identified fungal pathogens was always higher in the flooded wheat plants at the tillering stage, which further indicated a low resilience of the plant-associated mycobiota to hydrological stress at an early growth stage.

In addition, flooding affected also the fungal saprotrophic community, which represented the largest proportion of the characterized fungal functional guilds. Saprotrophs are less dependent on plants than other fungal groups, and most of their activity occurs around the rhizosphere because of the release of root exudates and other rhizodeposits. They promote mineralization processes, altering nutrient availability and may indirectly influence plant growth (Gams, 2007; Bardgett and van der Putten, 2014). This explains the large differences in saprotrophic community composition between the above and belowground compartments investigated herein. Fungal saprophytic communities of the root and rhizosphere compartments were particularly associated with shifts in root nutrient concentrations caused by flooding. This highlights the tight dependency of fungal saprotrophs with the soil environment. Indeed, saprophytes are expected to be more dependent upon their respective substrates than other fungal groups (Gebauer and Taylor, 1999) and could therefore be influenced by abiotic factors such as soil nutrients or soil moisture (Kubartová et al., 2009; Crowther et al., 2012; Francioli et al., 2021c). Furthermore, anoxia resulting from flooding may profoundly influence plant growth and thus indirectly alter the belowground mycobiota through changes in the quality and quantity of rhizodeposits, including exudates, competition for nutrients, or further mechanisms (Henry et al., 2007; Hartman and Tringe, 2019). Overall, changes in the composition of plant-associated mycobiota under flooding stress may have profound ecosystem-level effects on plant fitness and productivity, as well as on soil processes such as C, N and P cycles, within natural and agricultural ecosystems (Barnes et al., 2018).

Our study addressed for the first time all relevant plant compartments that are colonized by fungi and their response to flooding stress. Experiments under controlled glasshouse conditions represent an essential starting point, but there is a need to confirm such insights from controlled plant-level studies with field conditions that include a broader variance of soil parameters and weather as well as further biotic interactions. Research on flooding and waterlogging of the crop mycobiota in agroecosystems is limited to only a handful of studies. While the soil mycobiota is considerable resilient to drought (Bapiri et al., 2010; Kaisermann et al., 2017; de Vries et al., 2018), they are evidently highly sensitive to high soil moisture levels. This suggests that extreme precipitation that leads to waterlogging events represents an overlooked and important regulator of plant mycobiota assembly in agroecosystems under climatic threats. In summary, our findings support the adverse outlook of an increased plant pathogenicity under climate change scenarios in agricultural ecosystems.

## Data availability statement

The data presented in the study are deposited in the NCBI repository, accession number PRJNA902774.

## Author contributions

SK and M-RH planned the study. GC collected the samples. DF and GC performed the lab work, analyzed the data, and provided general guidance. DF wrote the manuscript. SK, M-RH, and GC contributed to reviewing the manuscript. All authors contributed to the article and approved the submitted version.

## Funding

This study was funded by the Leibniz Competition Program project “Volcorn-Volatilome of a Cereal Crop Microbiota Complex under Drought and Flooding” (K102/2018) (Leibniz Association).

## Acknowledgments

We thank Paul Reim and the technicians and gardeners of the IPK Gatersleben for their technical assistance during sampling and Kristina Holz (ZALF, Central Analytic Laboratory) for kindly providing the soil parameters. We thank Andreas Börner (IPK) for providing the seeds of the spring wheat cultivar Chinese Spring used in this study. We are also grateful to Caterina Maggi for her assistance.

## Conflict of interest

The authors declare that the research was conducted in the absence of any commercial or financial relationships that could be construed as a potential conflict of interest.

## Publisher’s note

All claims expressed in this article are solely those of the authors and do not necessarily represent those of their affiliated organizations, or those of the publisher, the editors and the reviewers. Any product that may be evaluated in this article, or claim that may be made by its manufacturer, is not guaranteed or endorsed by the publisher.
