# Supplementary material for: Response of the wheat mycobiota to flooding revealed substantial shifts towards plant pathogens
Source: Front Plant Sci. 2022 Nov 28;13:1028153. doi: 10.3389/fpls.2022.1028153 (PMC9742542; doi:10.3389/fpls.2022.1028153)
Supplement: Supplementary file 1 [file DataSheet_1.docx]

*Supplementary material*

Response of the wheat mycobiota to flooding revealed substantial shifts towards plant pathogens

Davide Francioli^1, 2^*, Geeisy Cid^3^, Mohammad-Reza Hajirezaei^3^ and Steffen Kolb^2, 4^

^1^Institute of Crop Science, Faculty of Agricultural Sciences, University of Hohenheim, Germany

^2^Microbial Biogeochemistry, Research Area Landscape Functioning, Leibniz Center for Agricultural Landscape Research e.V. (ZALF), Müncheberg, Germany

^3^Department of Physiology and Cell Biology, Leibniz Institute of Plant Genetics and Crop Plant Research, 06466 Gatersleben, Germany

^4^Thaer Institute, Faculty of Life Sciences, Humboldt University of Berlin, 10115 Berlin, Germany

* Corresponding author
*Davide Francioli
davide.francioli@zalf.de*

*Tel: +49 (0) 33432 82488*

TABLES

Table S1. Soil chemical properties of the soil used in the study (n=6).

| Parameter | Value | Mean standard error |
| --- | --- | --- |
| pH (KCl) | 5.42 | 0.003 |
| soil moisture | 3.43 | 0.232 |
| % TOC | 0.66 | 0.002 |
| % Nt | 0.07 | 0.001 |
| % St | 0.01 | 0.001 |
| NH4-N (mg/kg) | 0.78 | 0.003 |
| NO3-N (mg/kg) | 26.64 | 0.022 |
| PDL (mg/kg) | 60.80 | 0.011 |

Table S2. Shoot and root dry biomass, numbers of tiller and spike tiller ratio measured in the control and flooded samples (n=6). Values are means and letter denote significant differences between control and flooded treatment at each plant growth stages (Tukey’s HSD test, P < 0.05). Standard deviations are indicated in parentheses.

|  | Tillering | | Booting | | Flowering | |
| --- | --- | --- | --- | --- | --- | --- |
|  | Control | Flooding | Control | Flooding | Control | Flooding |
| Shoot dry weight (gr) | 2.04 (0.40) | 2.08 (0.61)- | 20.46 (2.61)A | 15.34 (1.85)B | 39.97 (2.62) | 41.12 (2.43) |
| Root dry weight (gr) | 2.18 (1.11)A | 0.80 (0.56)B | 5.89 (2.91)A | 1.63 (1.14)B | 10.61 (4.7) | 11.37 (3.49) |
| Number of tillers | - | - | 17.50 (2.51)A | 12.50 (2.33)B | 21 (1.92) | 21.12 (1.25) |
| Number of spikes | - | - | 2.12 (1.73) | 1.75 (1.58) | 19.25 (2.61)A | 16.75 (2.37)B |

Table S3. Soil and plant properties measured in the different water treatments and PGS. Values are means and letter denote significant differences between control and flooded treatment at each plant growth stages (Tukey’s HSD test, P < 0.05). Standard error of the means are indicated in parentheses.

|  | Tillering | | Booting | | Flowering | | |  |
| --- | --- | --- | --- | --- | --- | --- | --- | --- |
|  | Control | Flooding | Control | Flooding | Control | | Flooding | |
| *Rhizosphere* |  |  |  |  |  | |  | |
| soil pH | 6.76 (0.22)A | 7.69 (0.26)B | 6.61 (0.09)A | 7.77 (0.09)B | 6.52 (0.10)A | | 7.53 (0.16)B | |
| total organic C (%) | 0.762 (0.037)A | 0.824 (0.041)B | 0.768 (0.025) | 0.792 (0.041) | 0.838 (0.049) | | 0.84 (0.05) | |
| total N (%) | 0.083 (0.003) | 0.076 (0.011) | 0.084 (0.003) | 0.086 (0.005) | 0.074 (0.002)A | | 0.091(0.005)B | |
| total S (mg/Kg) | 98.2 (4.1)A | 107.8 (4.7)B | 101.4 (3.9) | 103.5 (8.2) | 115.6 (3.5) | | 115.5 (4.6) | |
| Available P (mg/Kg) | 63.14 (6.21)A | 92.76 (7.50)B | 63.19 (4.09)A | 95.18 (11.4)B | 62.75 (5.29)A | | 103.48 (8.25)B | |
| K (mg/Kg) | 1387 (311) | 1437 (141) | 1625 (451) | 1841 (281) | 1492 (256)A | 1790 (378)B | |  |
| Ca (mg/Kg) | 1472 (204) | 1456 (85.5) | 1496 (213)A | 1798 (207)B | 1567 (301) | 1580 (171) | |  |
| Na (mg/Kg) | 39.62 (13.97) | 50.92 (6.03) | 61.41 (22.85) | 69.37 (16.03) | 87.67 (30.01) | 82.5 (18.89) | |  |
| Mg (mg/Kg) | 1368 (52)A | 1243 (44)B | 1379 (154) | 1460 (100) | 1393 (268) | 1394.64 (85) | |  |
| Mn (mg/Kg) | 381.5 (12.5) | 390.5 (19.3) | 396.9 (25.6) | 401.43 (20.1) | 410.4 (26.4) | 400.6 (17.3) | |  |
|  |  |  |  |  |  |  | |  |
| *Root* |  |  |  |  |  |  | |  |
| C (%) | 37.08 (7.55) | 40.75 (1.59) | 41.05 (1.48) | 42.26 (2.06) | 40.84 (1.52) | 40.87 (0.92) | |  |
| N (%) | 1.03 (0.13) | 0.83 (0.26) | 2.30 (0.20)A | 1.66 (0.05)B | 1.72 (0.11) | 1.79 (0.08) | |  |
| P (mg/Kg) | 2470(482) | 2551 (487) | 2610 (137)A | 1387 (187)B | 1864 (100) | 1972 (168) | |  |
| S (mg/Kg) | 1376 (94.2) A | 1117 (172) B | 1616 (101) | 1501 (175) | 1720 (188) | 1785 (139) | |  |
| Mg (mg/Kg) | 1472 (330)A | 1046 (177)B | 2542 (266)A | 1337 (324)B | 1633 (244) | 1667.8 (133) | |  |
| Mn (mg/Kg) | 167.4 (76.4)A | 809.6 (245.9)B | 176.3 (31.6)A | 370.9 (91.1)B | 178.7 (27.6)A | 427.6 (36.1)B | |  |
| Na (mg/Kg) | 321 (97)A | 1236 (484)B | 2086 (422)A | 468 (228)B | 3068 (549) | 2807 (736) | |  |
| K (mg/Kg) | 13906 (3272) | 14545 (2182) | 8607 (785)A | 3784 (1069)B | 7463 (1394)A | 10543 (1140)B | |  |
|  |  |  |  |  |  |  | |  |
| *Leaf* |  |  |  |  |  |  | |  |
| C (%) | 41.91 (0.97)A | 43.99 (0.25)B | 45.77 (0.76)A | 46.95 (1.65)B | 45.77 (0.53) | 46.26 (0.51) | |  |
| N (%) | 1.51 (0.21)A | 0.94 (0.07)B | 4.52 (0.55) | 4.69 (0.34) | 3.46 (0.23) | 3.69 (0.38) | |  |
| P (mg/Kg) | 3568 (179)A | 1501 (379)B | 4332 (341)A | 3292 (203)B | 3042 (872) | 3345 (380) | |  |
| S (mg/Kg) | 1498 (129)A | 727 (61)B | 3500 (497)A | 2374 (345)B | 3319 (323) | 3248 (177) | |  |
| Mg (mg/Kg) | 673 (120)A | 450 (64)B | 2170 (343)A | 1329 (213)B | 2142 (89)A | 1966 (120)B | |  |
| Mn (mg/Kg) | 26.9 (7.7)A | 58.9 (9.2)B | 70.1 (16.3)A | 182.9 (62.8)B | 67.5 (13.7)A | 174.6 (12.9)B | |  |
| Ca (mg/Kg) | 2522 (437) | 2229 (527) | 6709 (2170) | 4811 (965) | 6478 (623) | 6621 (822) | |  |
| Na (mg/Kg) | 27.6 (7.7) A | 45.9 (8.3)B | 118.1 (16.8)A | 358.6 (85.2)B | 56.4 (21.2)A | 96.6 (15.2)B | |  |
| K (mg/Kg) | 29991 (1538)A | 12272 (2720)B | 26463 (5079)A | 21052 (4717)B | 23458 (2805) | 23072 (539) | |  |

Table S4. The relative importance of soil-plant compartment, plant growth stage (PGS) and watering treatment (WT) for the fungal richness in the samples investigated in this study. Only interactions with significant effects are reported.

| Parameter | df | Pseudo-F | R^2^ | P‐value |
| --- | --- | --- | --- | --- |
| Compartment | 1 | 542.45 | 0.7270 | 0.001 |
| PGS | 2 | 1.27 | 0.004 | 0.286 |
| WT | 1 | 11.15 | 0.041 | 0.001 |
| Compartment * WT | 2 | 3.93 | 0.006 | 0.038 |
| PGS * WT | 2 | 5.02 | 0.106 | 0.001 |

Table S5. List of the plant fungal pathogenic species identified in our study.

| Pathogenic species |
| --- |

*Gibberella intricans*

*Mycosphaerella gramicola*

*Truncatella angustata*

*Olpidium brassicae*

*Ilyonectria mors-panacis*

*Typhula sclerotioides*

*Bipolaris drechsleri*

*Gremmenia infestans*

*Blumeria graminis*

*Waitea circinata var. circinata*

*Typhula incarnata*

*Gaeumannomyces hyphopodioides*

*Curvularia lunata*

*Gaeumannomyces avenae*

*Eutypa maura*

*Ilyonectria robusta*

Table S6. The relative importance of soil-plant compartment, plant growth stage (PGS) and water treatment (WT) for the pathogenic richness associated to the samples investigated in this study. Only interactions with significant effects are reported.

|  |  | | | |
| --- | --- | --- | --- | --- |
| Parameter | df | Pseudo-F | R^2^ | P‐value |
| Compartment | 2 | 71.333 | 0.55499 | 0.001 |
| PGS | 2 | 5.644 | 0.04391 | 0.004 |
| WT | 1 | 1.099 | 0.00428 | 0.3 |
| Compartment * PGS | 4 | 3.159 | 0.04915 | 0.016 |

Table S7. The relative importance of soil-plant compartment, plant growth stage (PGS) and water treatment (WT) for the pathogenic community structure associated to the samples investigated in this study. Only interactions with significant effects are reported.

|  |  | | | |
| --- | --- | --- | --- | --- |
| Parameter | df | Pseudo-F | R^2^ | P‐value |
| Compartment | 2 | 41.686 | 0.3865 | 0.001 |
| PGS | 2 | 7.514 | 0.06966 | 0.001 |
| WT | 1 | 1.526 | 0.00707 | 0.183 |
| Compartment * PGS | 4 | 4.565 | 0.08464 | 0.001 |
| PGS * WT |  | 2.287 | 0.02121 | 0.035 |
| Compartment *PGS * WT |  | 1.871 | 0.03469 | 0.041 |

Table S8. The relative importance of soil-plant compartment, plant growth stage (PGS) and water treatment (WT) for the saprophytic richness associated to the samples investigated in this study. Only interactions with significant effects are reported.

|  |  | | | |
| --- | --- | --- | --- | --- |
| Parameter | df | Pseudo-F | R^2^ | P‐value |
| Compartment | 2 | 711.38 | 0.89197 | 0.001 |
| PGS | 2 | 0.82 | 0.00103 | 0.414 |
| WT | 1 | 9.27 | 0.00581 | 0.005 |
| Compartment * WT | 2 | 4.07 | 0.0051 | 0.021 |
| Compartment *PGS | 4 | 3.26 | 0.00817 | 0.012 |
| PGS * WT | 2 | 5.07 | 0.00636 | 0.011 |
| Compartment *PGS * WT | 4 | 11.53 | 0.02891 | 0.001 |

Table S9. The relative importance of soil-plant compartment, plant growth stage (PGS) and water treatment (WT) for the saprophytic community structure associated to the samples investigated in this study. Only interactions with significant effects are reported.

|  |  | | | |
| --- | --- | --- | --- | --- |
| Parameter | df | Pseudo-F | R^2^ | P‐value |
| Compartment | 2 | 59.362 | 0.4962 | 0.001 |
| PGS | 2 | 3.469 | 0.029 | 0.002 |
| WT | 1 | 2.501 | 0.01045 | 0.022 |
| Compartment * WT | 2 | 2.426 | 0.02028 | 0.014 |
| Compartment *PGS | 4 | 2.799 | 0.04679 | 0.001 |
| PGS * WT | 2 | 2.027 | 0.01694 | 0.02 |
| Compartment *PGS * WT | 4 | 1.751 | 0.02927 | 0.022 |

Table S10. The relative importance of soil-plant compartment, plant growth stage (PGS) and water treatment (WT) for the mutualist richness associated to the samples investigated in this study. Only interactions with significant effects are reported. Solely the mutualistic taxa detected in the rhizosphere and root compartments were included in this analysis since no mutualist taxa were detected in the phyllosphere.

|  |  | | | |
| --- | --- | --- | --- | --- |
| Parameter | df | Pseudo-F | R^2^ | P‐value |
| Compartment | 2 | 93.314 | 0.61439 | 0.001 |
| PGS | 1 | 9.378 | 0.03087 | 0.003 |
| WT | 2 | 0.426 | 0.0028 | 0.635 |
| Compartment * WT | 2 | 6.279 | 0.04134 | 0.007 |

Table S11. The relative importance of soil-plant compartment, plant growth stage (PGS) and water treatment (WT) for the mutualist community structure associated to the samples investigated in this study. Only interactions with significant effects are reported. Solely the mutualistic taxa detected in the rhizosphere and root compartments were included in this analysis since no mutualist taxa were detected in the phyllosphere.

|  |  | | | |
| --- | --- | --- | --- | --- |
| Parameter | df | Pseudo-F | R^2^ | P‐value |
| Compartment | 1 | 15.2162 | 0.17717 | 0.001 |
| PGS | 2 | 1.411 | 0.03286 | 0.045 |
| WT | 1 | 1.9824 | 0.02308 | 0.023 |
| Compartment * PGS | 2 | 2.0885 | 0.04864 | 0.006 |
| WT * PGS | 2 | 1.5975 | 0.03748 | 0.04 |

Table S12 Relationships between the predictor soil and plant properties and the fungal pathogenic communities in the leaf, rhizosphere and root compartments. Results show marginal tests using the db-RDA model. Significant P-values less than 0.05 are indicated in bold. ns, no significant.

|  | Leaf | | Rhizosphere | | | Root | |  |
| --- | --- | --- | --- | --- | --- | --- | --- | --- |
|  | F | P | | F | P | F | P | |
| Soil pH | ns | ns | | ns | ns | **3.609** | **0.004** | |
| Soil K | ns | ns | | ns | ns | **4.069** | **0.002** | |
| Soil S | ns | ns | | **2.403** | **0.023** | **3.928** | **0.001** | |
| Root C | **2.672** | **0.043** | | ns | ns | ns | ns | |
| Root S | **2.987** | **0.023** | | ns | ns | ns | ns | |
| Leaf Na | **2.874** | **0.03** | | ns | ns | ns | ns | |
| Leaf Mn | **5.886** | **0.001** | | ns | ns | ns | ns | |

Table S13 Relationships between the predictor soil and plant properties and the fungal saprophytic communities in the leaf, rhizosphere and root compartments. Results show marginal tests using the db-RDA model. Significant P-values less than 0.05 are indicated in bold. ns, no significant.

|  | Leaf | | Rhizosphere | | | Root | | |  |
| --- | --- | --- | --- | --- | --- | --- | --- | --- | --- |
|  | F | P | | F | P | | F | P | |
| Soil pH | ns | ns | | **3.992** | **0.001** | | ns | ns | |
| Root C | **2.244** | **0.001** | | **1.954** | **0.002** | | **1.724** | **0.013** | |
| Root P | ns | ns | | ns | ns | | **1.979** | **0.001** | |
| Root Mn | ns | ns | | **1.505** | **0.041** | | ns | ns | |
| Root Mg | ns | ns | | **1.465** | **0.034** | | **1.468** | **0.05** | |
| Leaf S | **4.617** | **0.001** | | ns | ns | | ns | ns | |
| Leaf Na | **1.786** | **0.016** | | ns | ns | | ns | ns | |

Table S14 Relationships between the predictor soil and plant properties and the fungal mutualist communities in the rhizosphere and root compartments. Results show marginal tests using the db-RDA model. Significant P-values less than 0.05 are indicated in bold. ns, no significant. PDL, plant available P.

|  | Rhizosphere | | Root | | |
| --- | --- | --- | --- | --- | --- |
|  | F | P | F | P |  |
| Soil pH | **1.52** | **0.029** | ns | ns |  |
| PDL | **1.43** | **0.047** | ns | ns |  |
| Root S | **2.21** | **0.001** | ns | ns |  |
| Root K | ns | ns | **3.05** | **0.001** |  |
| Root Na | ns | ns | **1.48** | **0.046** |  |

**FIGURE S1 Experimental design of the greenhouse experiment conducted in this study (edited from Francioli et al. (2021a)).**


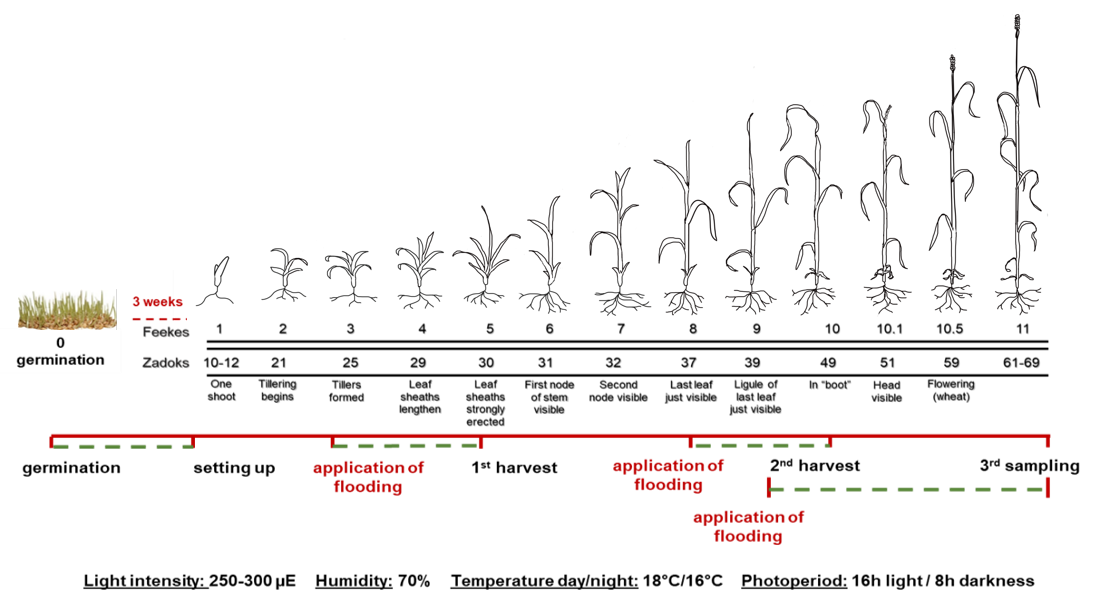


**FIGURE S2. LEfSe analysis at multiple taxonomic levels comparing fungal communities associated with leaf, rhizosphere and root of spring wheat plants. Cladogram illustrating the taxonomic groups explaining the most variation among the fungal communities. Each ring represents a taxonomic level, with phylum (p), class (c) and order (o) emanating from the center to the periphery. Each circle is a taxonomic unit found in the dataset, with circles or nodes shown in colors (other than yellow) indicating where a taxon was significantly more abundant.**

**
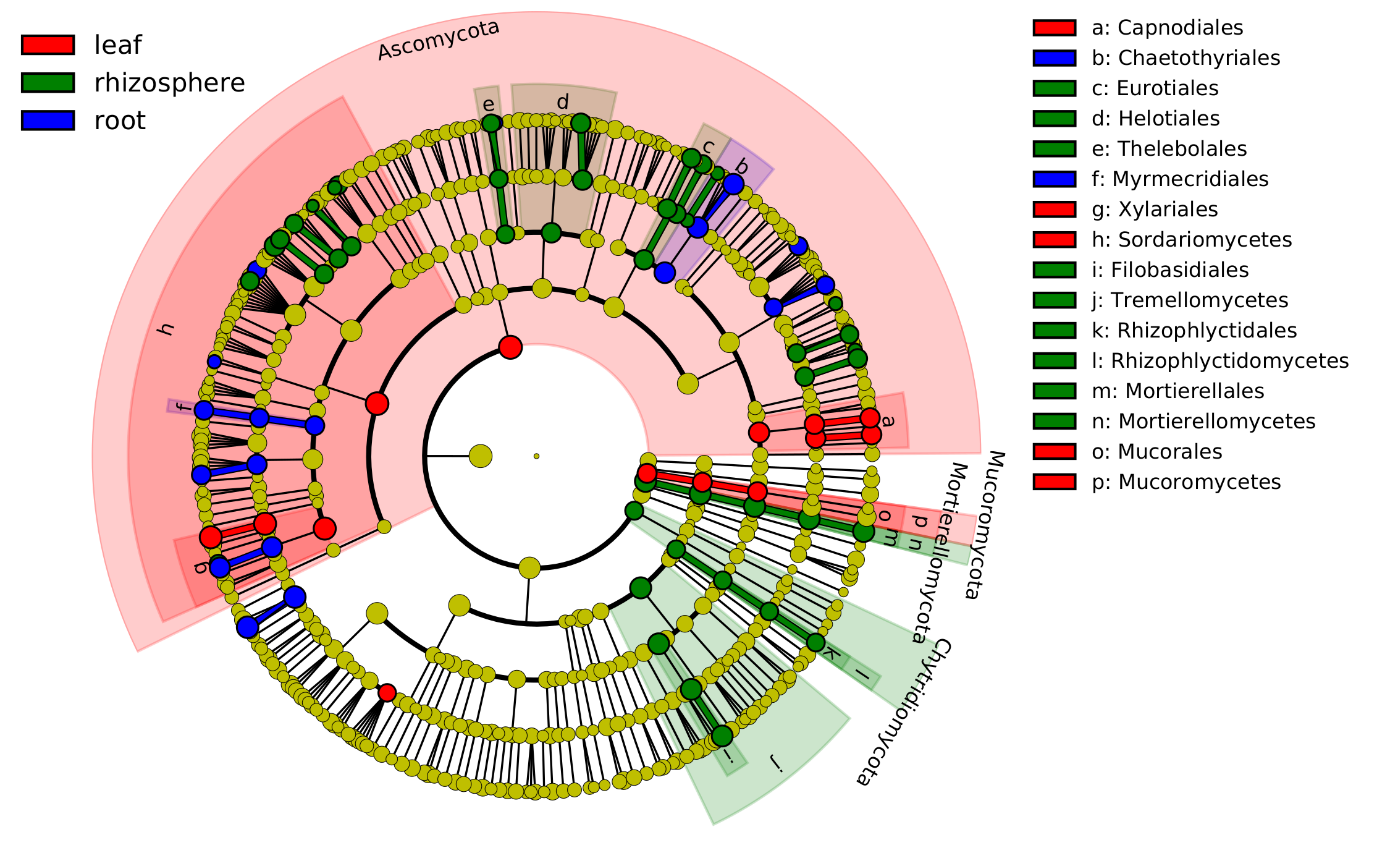
**

**FIGURE S3. LEfSe analysis at multiple taxonomic levels comparing leaf fungal community structure in both control and flooding treatment at tillering (A), booting (B) and flowering (C). Cladogram illustrating the taxonomic groups explaining the most variation among root communities. Each ring represents a taxonomic level, with phylum (p), class (c), order (o), family (f) and genus (g) emanating from the center to the periphery. Each circle is a taxonomic unit found in the dataset, with circles or nodes shown in colors (other than yellow) indicating where a taxon was significantly more abundant.**


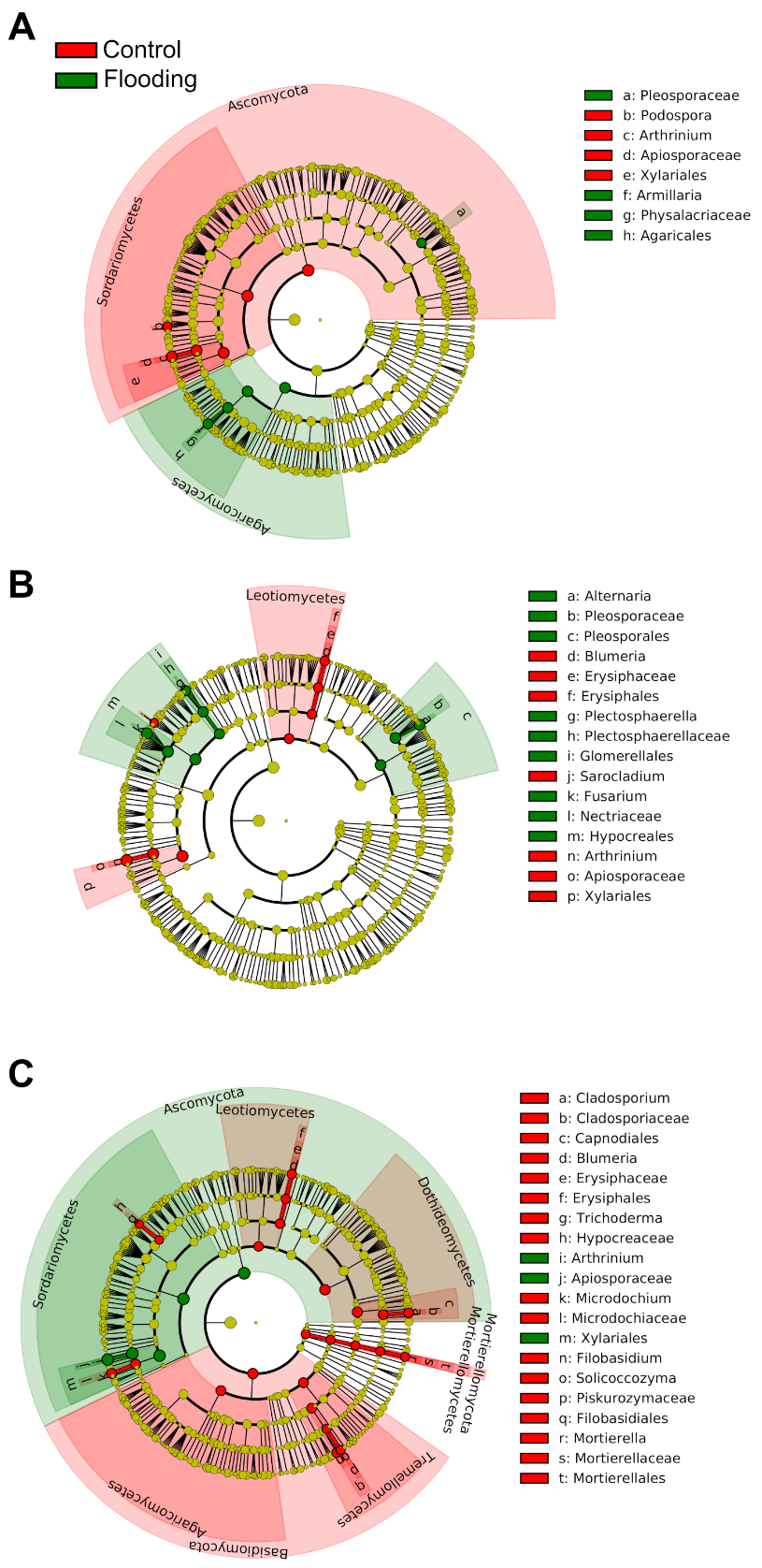


**FIGURE S4. LEfSe analysis at multiple taxonomic levels comparing rhizospheric fungal community structure in both control and flooding treatment at tillering (A), booting (B) and flowering (C). Cladogram illustrating the taxonomic groups explaining the most variation among root communities. Each ring represents a taxonomic level, with phylum (p), class (c), order (o), family (f) and genus (g) emanating from the center to the periphery. Each circle is a taxonomic unit found in the dataset, with circles or nodes shown in colors (other than yellow) indicating where a taxon was significantly more abundant.**

**
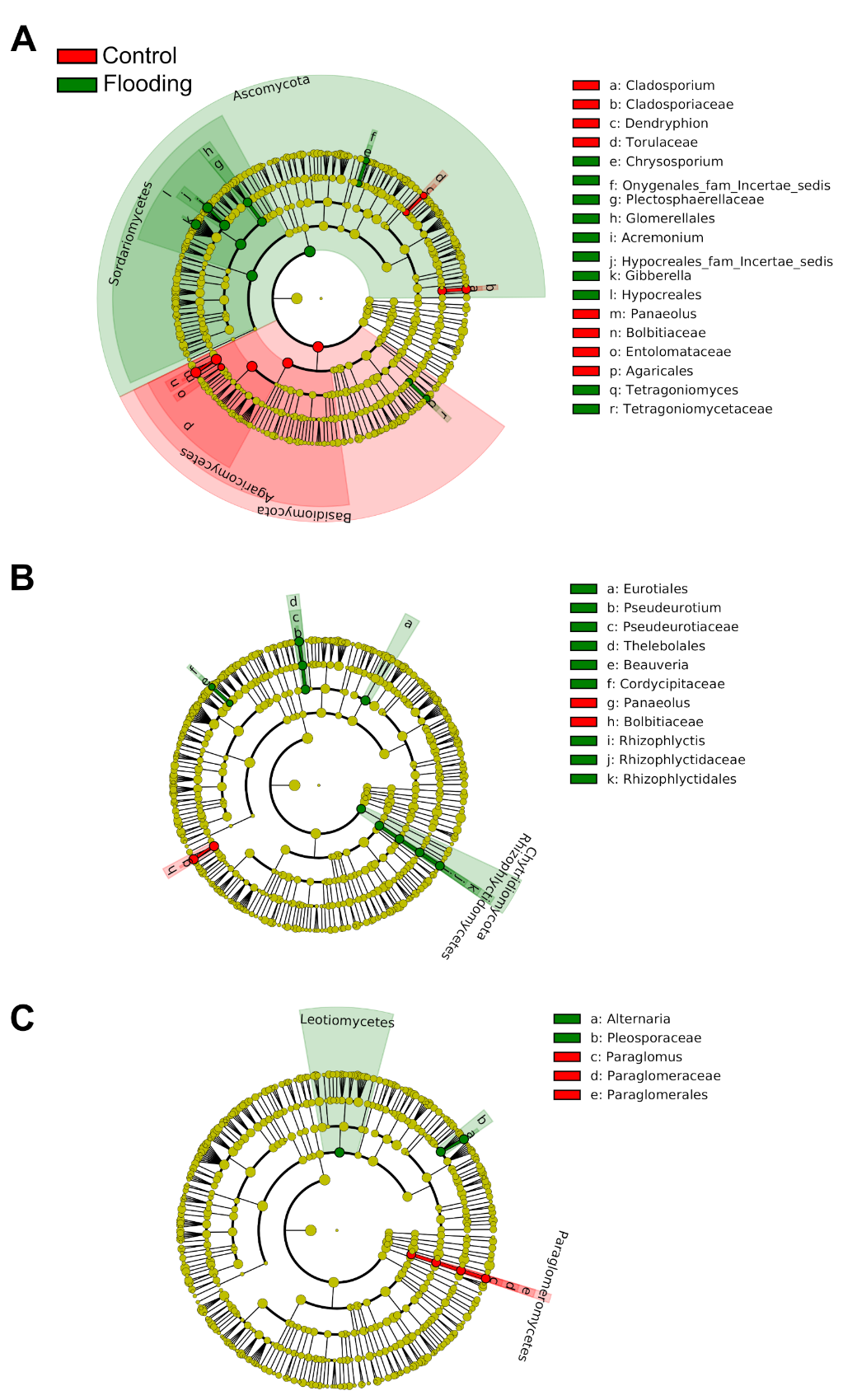
**

**FIGURE S5. LEfSe analysis at multiple taxonomic levels comparing root fungal community structure in both control and flooding treatment at tillering (A), booting (B) and flowering (C). Cladogram illustrating the taxonomic groups explaining the most variation among root communities. Each ring represents a taxonomic level, with phylum (p), class (c), order (o), family (f) and genus (g) emanating from the center to the periphery. Each circle is a taxonomic unit found in the dataset, with circles or nodes shown in colors (other than yellow) indicating where a taxon was significantly more abundant.**

**
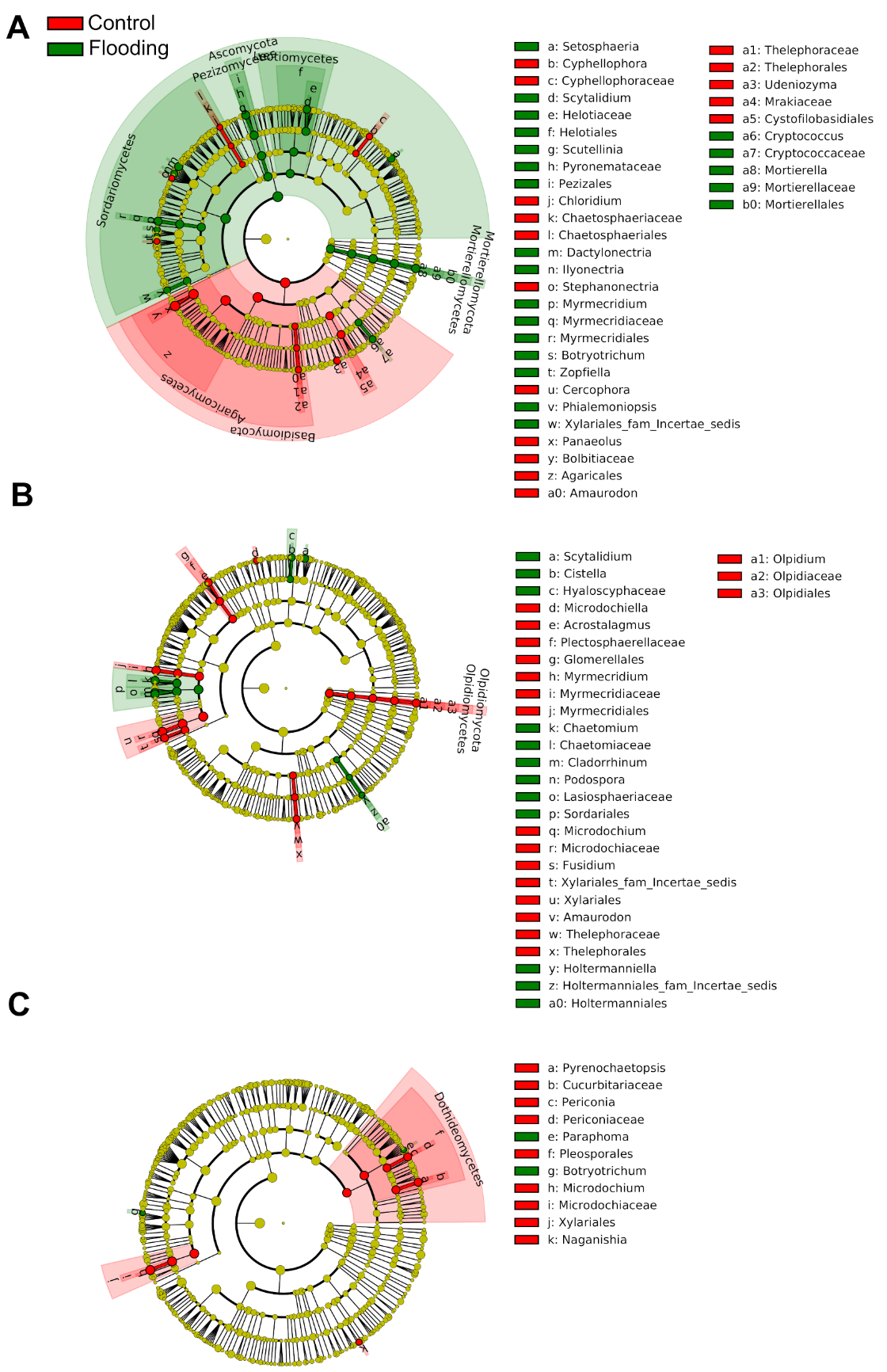
**

**FIGURE S6.** Box plots of the observed fungal pathogenic richness across soil-plant compartments, plant growth stages (PGS) and water treatment.

**
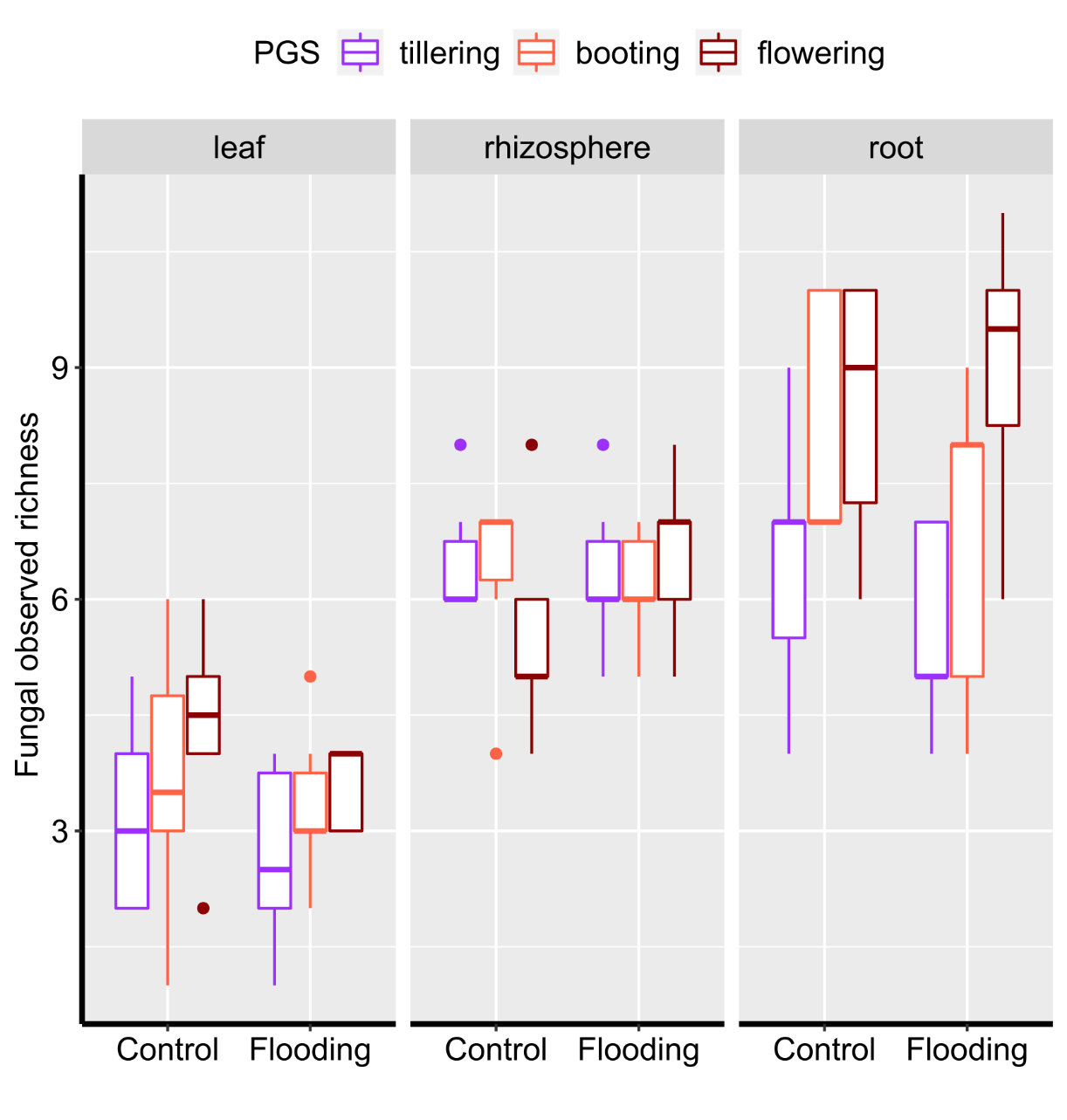
**

**FIGURE S7.** **Principal Coordinates Analysis (PCoA) of the fungal pathogenic community structures detected in this study.**


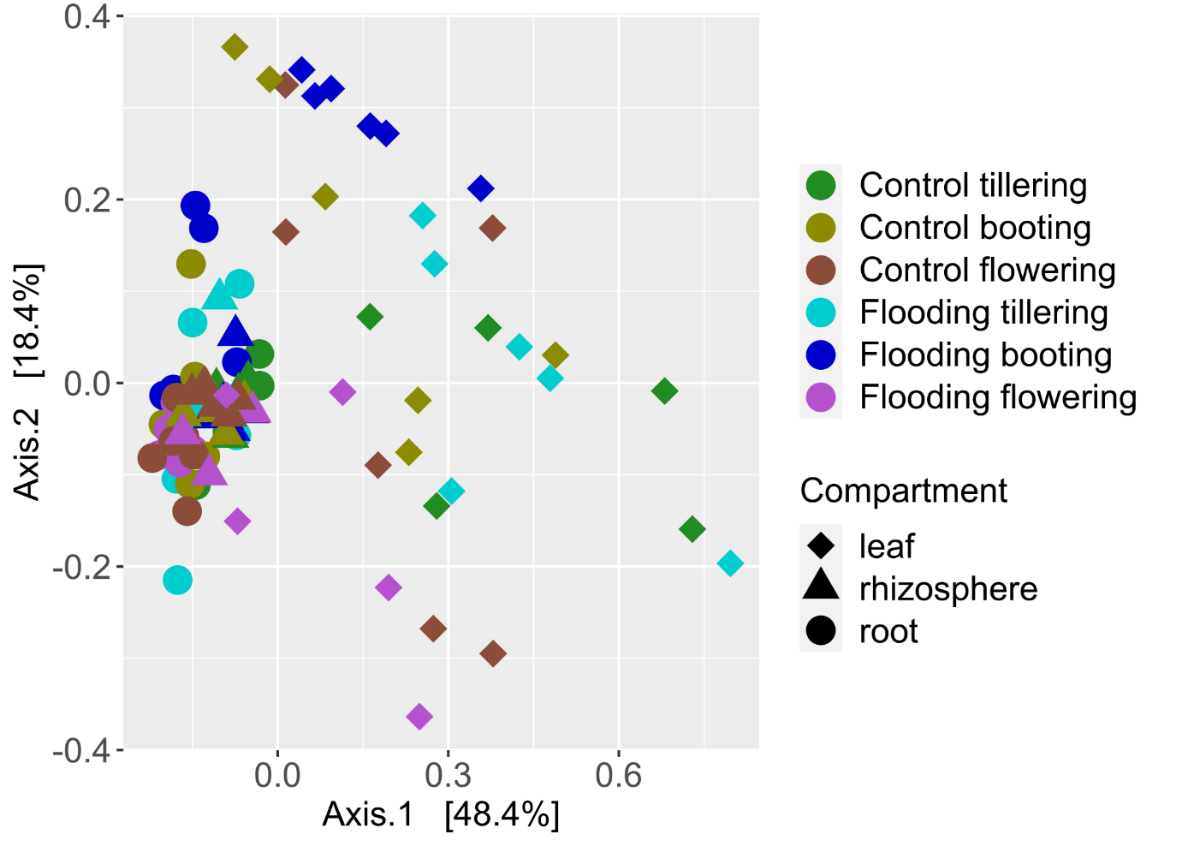


**FIGURE S8.** Box plots of the observed fungal **saprophytic** richness across soil-plant compartments, plant growth stages (PGS) and water treatment.


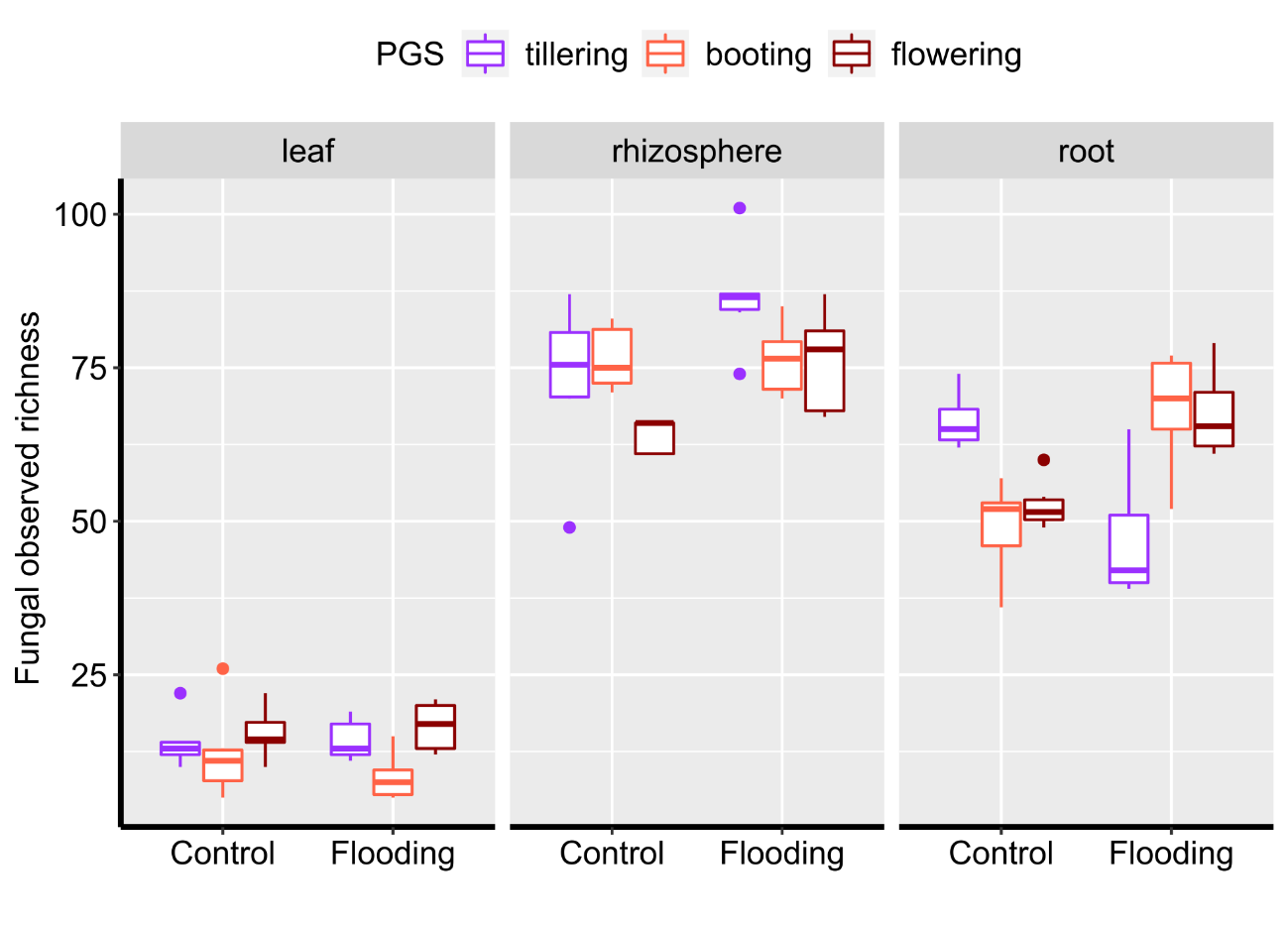


**FIGURE S9.** **Principal Coordinates Analysis (PCoA) of the fungal saprophytic communities detected in this study.**

**
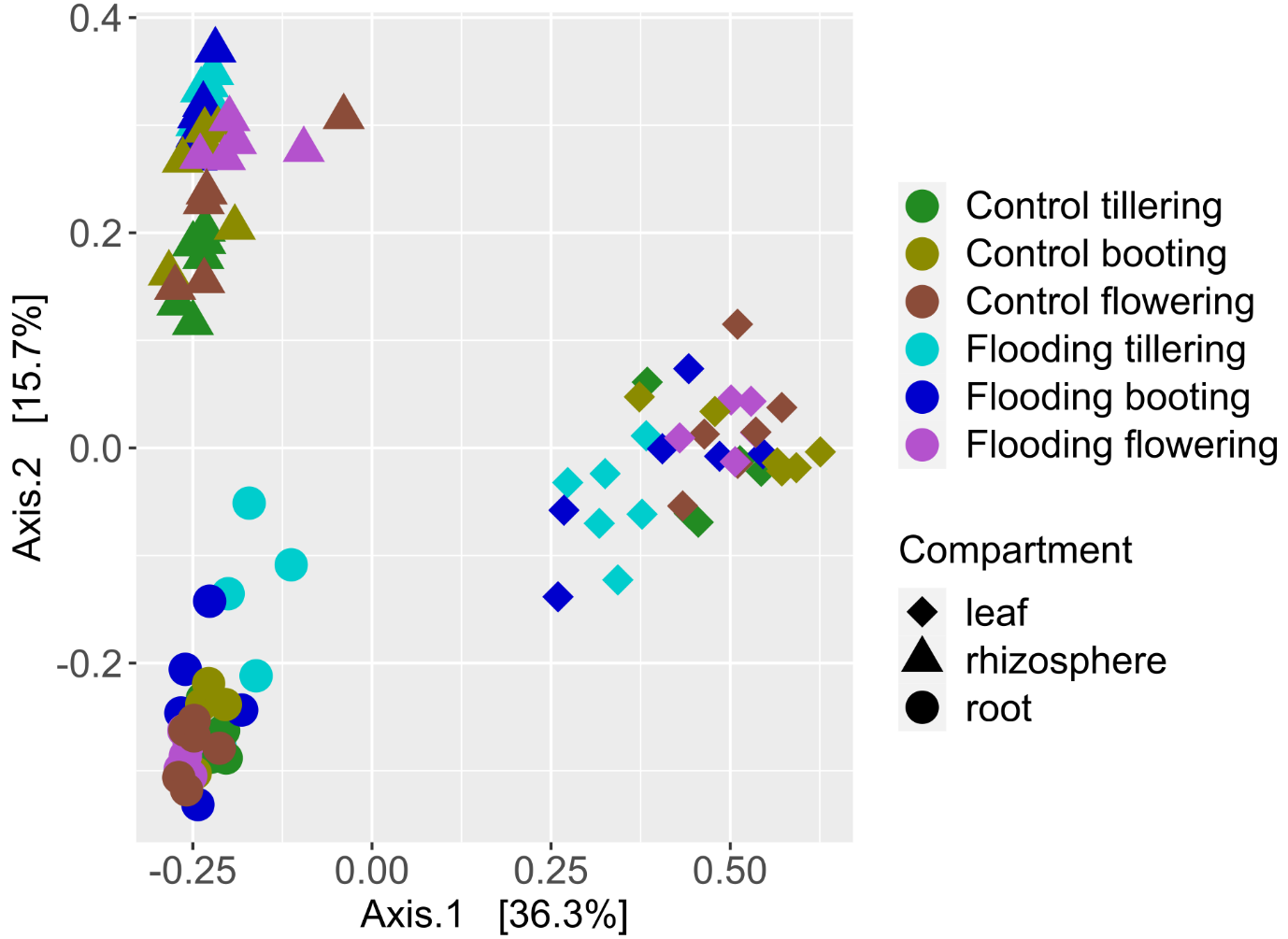
**

**FIGURE S10.** **Principal Coordinates Analysis (PCoA) of the fungal mutualist communities detected in this study.**

**
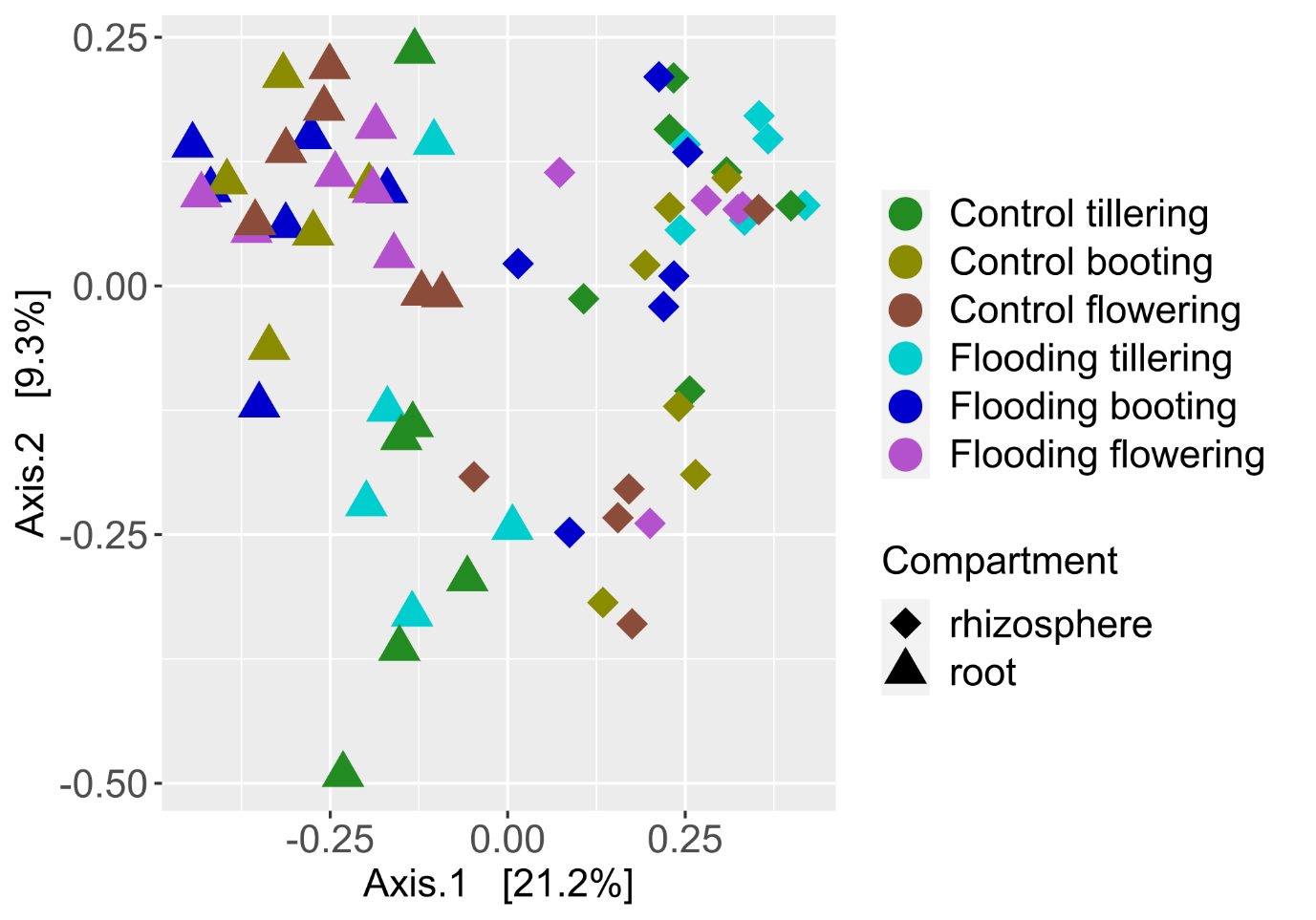
**

**FIGURE S11.** Box plots of the observed fungal mutualist richness across soil-plant compartments, plant growth stages (PGS) and water treatment.


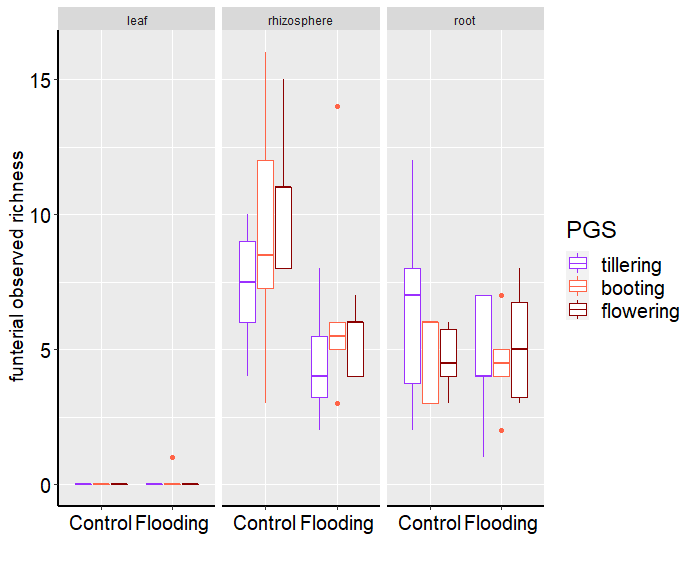


FIGURE S12. Variation partitioning analysis illustrating the effects soil parameters, plant physiological traits, watering treatment and plant growth stage (PGS) on the fungal functional guilds. Pathogenic fungal community of the (a) leaf, (b) rhizosphere and (c) root. Saprophytic fungal community of the (d) leaf, (e) rhizosphere and (f) root. Mutualistic fungal community of the (g) rhizosphere and (h) root.

**
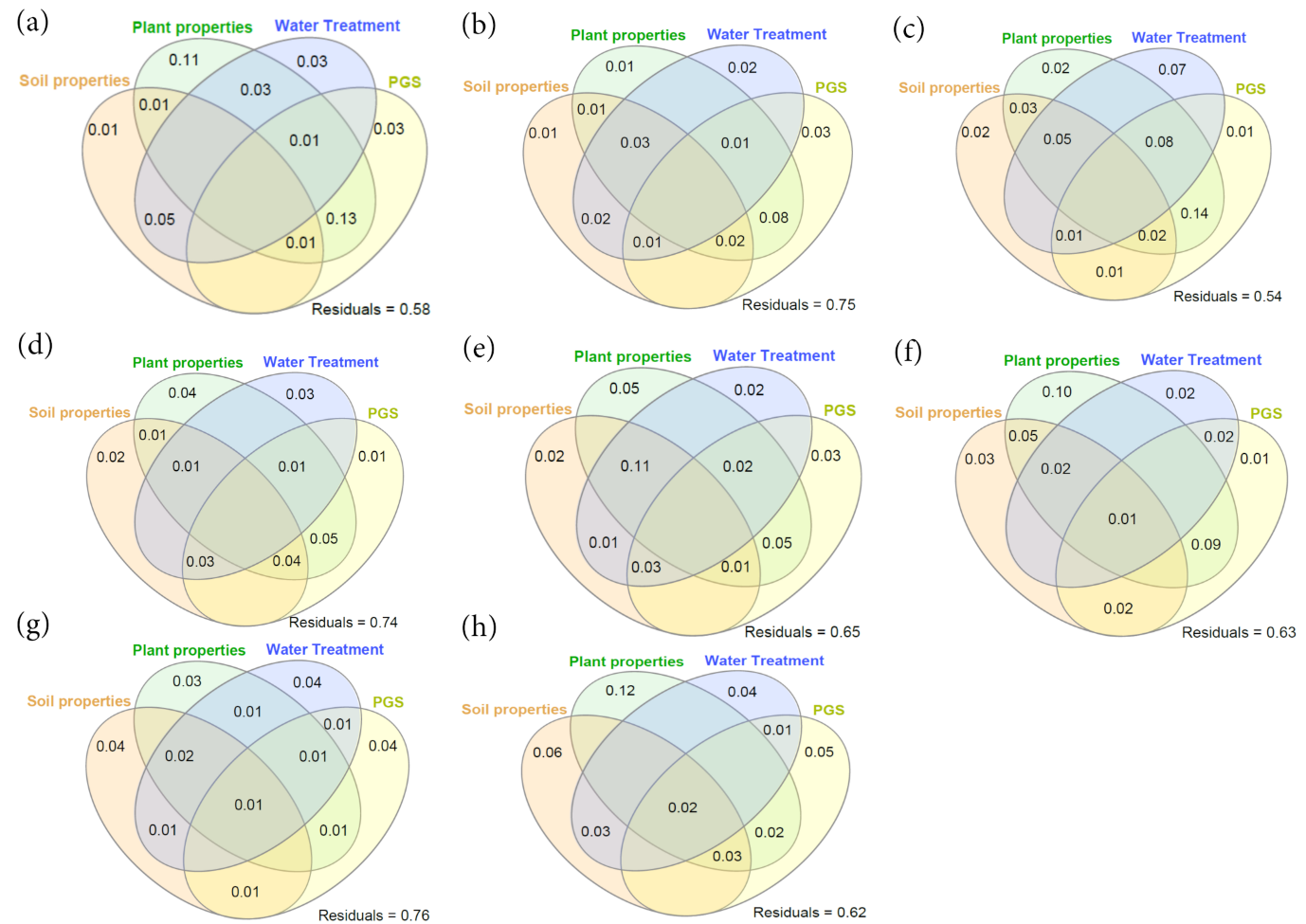
**
